# Selective inhibition of ASIC1a confers functional and morphological neuroprotection following traumatic spinal cord injury

**DOI:** 10.12688/f1000research.9094.2

**Published:** 2016-12-07

**Authors:** Liam M. Koehn, Natassya M. Noor, Qing Dong, Sing-Yan Er, Lachlan D. Rash, Glenn F. King, Katarzyna M. Dziegielewska, Norman R. Saunders, Mark D. Habgood

**Affiliations:** 1 Department of Pharmacology and Therapeutics, University of Melbourne, Parkville, Australia; 2 Division of Chemistry and Structural Biology, Institute for Molecular Bioscience, The University of Queensland, St. Lucia, Australia; 3 School of Biomedical Sciences, The University of Queensland, St. Lucia, Australia

**Keywords:** Spinal trauma, Neuroprotection, Psalmotoxin, PcTx1, Blood-spinal cord barrier, Acid-sensing ion channel, Ischaemia, Transcriptomic

## Abstract

Tissue loss after spinal trauma is biphasic, with initial mechanical/haemorrhagic damage at the time of impact being followed by gradual secondary expansion into adjacent, previously unaffected tissue. Limiting the extent of this secondary expansion of tissue damage has the potential to preserve greater residual spinal cord function in patients. The acute tissue hypoxia resulting from spinal cord injury (SCI) activates acid-sensing ion channel 1a (ASIC1a). We surmised that antagonism of this channel should provide neuroprotection and functional preservation after SCI. We show that systemic administration of the spider-venom peptide PcTx1, a selective inhibitor of ASIC1a, improves locomotor function in adult Sprague Dawley rats after thoracic SCI. The degree of functional improvement correlated with the degree of tissue preservation in descending white matter tracts involved in hind limb locomotor function. Transcriptomic analysis suggests that PcTx1-induced preservation of spinal cord tissue does not result from a reduction in apoptosis, with no evidence of down-regulation of key genes involved in either the intrinsic or extrinsic apoptotic pathways. We also demonstrate that trauma-induced disruption of blood-spinal cord barrier function persists for at least 4 days post-injury for compounds up to 10 kDa in size, whereas barrier function is restored for larger molecules within a few hours. This temporary loss of barrier function provides a “ *treatment window*” through which systemically administered drugs have unrestricted access to spinal tissue in and around the sites of trauma. Taken together, our data provide evidence to support the use of ASIC1a inhibitors as a therapeutic treatment for SCI. This study also emphasizes the importance of objectively grading the functional severity of initial injuries (even when using standardized impacts) and we describe a simple scoring system based on hind limb function that could be adopted in future studies.

## Introduction

Traumatic spinal cord injuries (SCIs) are devastating for patients due to the sudden and irreversible loss of motor, sensory and autonomic functions at and below the level of injury ( Hou & Rabchevsky, 2014; Scivoletto *et al.*, 2014). It is estimated that as many as 500,000 people suffer a spinal cord injury every year ( World Health Organization, 2013) with each incident requiring lifelong medical care amounting to approximately $USD 3.5–6.8 million over the course of a lifetime. There are currently *no effective pharmacological treatments* available to reverse trauma-induced tissue loss and restore lost functions for patients. The prevalence of this devastating and currently untreatable injury makes it a prominent issue for biomedical research.

The extent of functional losses following a SCI is largely determined by two factors: (i) the level at which the injury occurs (tetraplegia with cervical injuries or paraplegia with thoracic and lumbar injuries) and (ii) the extent of tissue damage at the lesion site (complete or incomplete). The pathology of tissue damage after SCI occurs in a biphasic manner. Initial physical (primary) damage at the time of injury due to mechanical compression, stretching and shearing of tissue is typically localised to the central grey matter ( Ek *et al.*, 2010; Schwab *et al.*, 2006; Tator & Fehlings, 1991; Wolman, 1965). This is followed by a period of ‘secondary’ expansion of the lesion into surrounding undamaged tissue (the peri-injury zone) over hours to days after injury ( Ek *et al.*, 2012; Zhang *et al.*, 2012). Our previous work has shown that secondary loss of grey matter is mostly complete within the first 24 h, whereas secondary loss of surrounding white matter tracts continues for several days post-injury ( Ek *et al.*, 2010; Ek *et al.*, 2012). Since this secondary loss is tissue that survived the initial impact, it is potentially salvageable if suitable neuroprotective treatments can be identified, administered and gain access to the injury site before it has become irreversibly damaged.

Primary tissue loss is characterized by extensive necrotic cell death similar to haemorrhagic stroke. Indeed, magnetic resonance imaging (MRI) studies of human SCIs have shown positive correlations between the extent of spinal haemorrhage/oedema and the extent of permanent functional deficits in patients ( Boldin *et al.*, 2006; Flanders *et al.*, 1999; Parashari *et al.*, 2011). In contrast, secondary tissue loss largely occurs by apoptotic cell death ( Byrnes *et al.*, 2007; Yong *et al.*, 1998). The mechanisms underlying this secondary apoptotic period are thought to involve structural, cellular, biochemical and vascular changes in the region surrounding the primary injury site (reviewed in Schwartz & Fehlings, 2002 and Zhang *et al.*, 2012). Vascular compromise appears to play a prominent role, with evidence from animal and human studies showing marked reductions in blood flow at the site of the spinal injury and in adjacent proximal regions ( Rivlin & Tator, 1978; Soubeyrand *et al.*, 2013; Tei *et al.*, 2005). Compression and rupture of central grey matter blood vessels not only disrupts blood supply to the central grey matter, but also to the deeper layers of surrounding white matter that are supplied by these damaged central vessels ( Koyanagi *et al.*, 1993; Losey & Anthony, 2014; Losey *et al.*, 2014; Tator & Koyanagi, 1997). The resulting hypoperfusion creates a zone of acute tissue hypoxia and ischaemia surrounding the physical lesion, commonly referred to as an ischemic penumbra. Trauma-induced ischaemia, and the consequent hypoxia, are widely regarded as central initiators of the cascade of events underlying secondary tissue damage after SCI ( Amar & Levy, 1999; Rowland *et al.*, 2008; Tator & Fehlings, 1991; Tator & Koyanagi, 1997) and have also been shown to promote apoptosis in rats ( Linnik *et al.*, 1993) and piglets ( Mehmet *et al.*, 1994).

The mechanisms by which hypoxia/ischaemia induce cell death are not clearly understood. Acute tissue acidosis in regions of hypoperfusion is sufficient to activate acid-sensing ion channel 1a (ASIC1a), a proton-gated ion channel that mediates influx of sodium and calcium into cells ( Yermolaieva *et al.*, 2004). Preventing ASIC1a activation with intravenous HCO _3_^-^ reduced tissue loss and functional deficits in a traumatic brain injury model ( Yin *et al.*, 2013), and genetic ablation or pharmacological inhibition of ASIC1a reduces neuronal injury following ischemic stroke ( Xiong *et al.*, 2004; Yin *et al.*, 2013). It has been proposed that excessive Ca ^2+^ influx via ASIC1a may induce mitochondrial dysfunction ( Friese *et al.*, 2007; Sherwood *et al.*, 2011) and promote activation of intrinsic apoptotic pathways including alterations in BAX/BCL2 ratios and activation of caspase-3 ( Smaili *et al.*, 2003).

ASIC1a is expressed by most neurons in the central and peripheral nervous systems and multiple studies have confirmed expression on spinal cord neurons ( Baron *et al.*, 2008; Wu *et al.*, 2004). ASIC1a expression on oligodendrocyte lineage cells has also been reported, implicating this channel in both grey and white matter damage ( Feldman *et al.*, 2008).

Psalmotoxin (PcTx1) is a 40-residue, 4.6 kDa peptide from venom of the Trinidad chevron tarantula, *Psalmopoeus cambridgei* ( Escoubas *et al.*, 2000). PcTx1 is the most potent described inhibitor of ASIC1a with an IC _50_ of ~1 nM, and it has minimal effect on other ASIC subtypes ( Escoubas *et al.*, 2000). In addition to PcTx1, ASIC1a is inhibited to a lesser extent by the diuretic amiloride (IC _50_ ~10 μM; ( Gründer & Chen, 2010)), non-steroidal anti-inflammatory drugs such as flurbiprofen (IC _50_ ~350 μM; ( Voilley *et al.*, 2001)) and alkaloids such as sinomenine (IC _50_ ~0.27 μM; ( Wu *et al.*, 2011)). While all of these non-selective ASIC blockers are neuroprotective in rodent stroke models ( Mishra *et al.*, 2011; Wu *et al.*, 2011; Xiong *et al.*, 2004; Zheng *et al.*, 2007), PcTx1 provides the best level of neuroprotection ( Pignataro *et al.*, 2007; Xiong *et al.*, 2004; Xiong *et al.*, 2006; Xiong *et al.*, 2008). Intracerebroventricular administration of recombinant PcTx1 at 2 h post-stroke was found to reduce infarct volume by ~70% in a rat model of middle cerebral artery occlusion, which correlated with improvements in neurological score and motor function as well as preservation of neuronal architecture ( McCarthy *et al.*, 2015). There has been one report of neuroprotection in a SCI model using intrathecal administration of a *P. cambridgei* venom extract containing PcTx1 ( Hu *et al.*, 2011). Unfortunately, PcTx1 constitutes only ~0.4% of the protein content of *P. cambridgei* venom ( McCarthy *et al.*, 2015), which contains hundreds of other bioactive peptides that act on a range of ligand- and voltage-gated ion channels. Thus, the results obtained with crude venom extracts cannot be taken as definitive evidence that ASIC1a antagonism by PcTx1 is neuroprotective in SCI.

Here we show conclusively that pure recombinant PcTx1 delivered systemically is neuroprotective after SCI in rats, reducing the extent of secondary tissue loss and improving locomotor function. In addition, transcriptomic analysis revealed a significant effect of initial lesion size on the differential expression of genes in different regions of the spinal cord in relation to the injury centre. Inflammatory associated genes predominated in the rostral penumbra, whereas genes associated with blood vessels ( *e.g.* actin and myosin) dominated at the lesion centre, possibly indicative of vascular remodelling. Together the transcriptomic data suggest that the mechanism of action by which PcTx1 confers neuroprotection involves modification of inflammatory and vascular responses to SCI.

## Materials and methods

### Production of recombinant PcTx1

Recombinant PcTx1 was produced as described previously via expression in the periplasm of *Escherichia coli* ( Saez *et al.*, 2011; Saez *et al.*, 2015). Briefly, recombinant His _6_-MBP-PcTx1 fusion protein was isolated from cell lysates by passage over Ni-NTA Superflow resin (QIAGEN) and the His _6_-MBP tag was then removed by cleavage of the fusion protein with tobacco etch virus (TEV) protease. The released recombinant PcTx1 containing a non-native N-terminal serine to facilitate TEV cleavage ( Saez *et al.*, 2011) was isolated to >95% purity using reverse-phase HPLC. The isolated recombinant PcTx1 peptide is equipotent with native PcTx1 ( Saez *et al.*, 2011).

### Animals

All procedures involving animals were approved by The University of Melbourne Animal Ethics Committee (Approval number: 1212637) and conducted in compliance with Australian National Health and Medical Research guidelines. Adult female Sprague Dawley rats (weight range 205–285g) were supplied by The University of Melbourne Biological Research Facility and housed in groups of 2–4 per cage on a 12 h light/dark cycle with *ad libitum* access to food and water. A total of 72 rats were randomly assigned into four experimental groups; PcTx1-treated ( *n*=12), saline-treated ( *n*=16), uninjured/untreated controls ( *n*=6) or blood-spinal cord barrier integrity ( *n*=38). 

### Spinal contusion injuries

Rats were deeply anaesthetized with inhaled isoflurane (3% in oxygen at 1.5 L/min, Lyppard Australia). The thoracic spinal cord was exposed at the T10 vertebral level via a skin incision and vertebral laminectomy. The vertebral column was then stabilized in a stereotaxic frame with clamps attached to the T9 and T11 dorsal vertebral spines. A single contusion injury was applied to the dorsal surface of the exposed spinal cord using a computer-controlled impactor ( Ek *et al.*, 2010; Ek *et al.*, 2012) using impact parameters previously determined to produce moderate incomplete spinal cord lesions mostly confined to the central grey matter.

### Treatment protocols

Mini-osmotic pumps (Alzet ^®^1003D, BioScientific, Australia) were filled with a solution of PcTx1 in sterile saline (1.03 mg/ml) and primed for 12–24 h at 37°C in phosphate buffered saline (PBS). Immediately after the contusion injury, animals assigned to the PcTx1-treated group ( *n*=12) received an intraperitoneal loading dose of PcTx1 (12.5 μg/kg) and a mini-osmotic pump containing PcTx1 was implanted subcutaneously between the shoulder blades and the wound site closed with several layers of sutures. The role of the pump was to slowly and steadily release PcTx1 (1.08 μg/h) subcutaneously to compensate for estimated renal and tissue losses of PcTx1 over the first 48 h period. Saline-treated animals ( *n*=16) received an equivalent intraperitoneal injection of saline, but no osmotic pump was implanted. Uninjured, untreated rats ( *n*=6) were included as reference controls.

### Assessing and grading the severity of the initial injuries

An inherent feature of all spinal contusion models is inter-animal variations in the size of the initial spinal cord lesions produced. Whilst the impactor device used in this study delivers standardized and highly reproducible impacts ( Ek *et al.*, 2010; Ek *et al.*, 2012), differences in the number and location of ruptured blood vessels will result in differences in the extent of tissue ischaemia, hypoxia and ultimately tissue loss. Accordingly, the functional severity of the initial injuries in each animal was assessed upon full recovery from anaesthesia and again at 24 h post-injury using a simplified injury severity (SIS) scale ranging from 0 (normal locomotion) to 3 (complete flaccid hind limb paralysis; see Table 2). This scale was independently developed, but the assessment criteria are similar to those described by Herrmann *et al.* (2008) and Anderson *et al.* (2016), and shown in Table 2. Each hind limb was assessed separately and an average of both hind limbs recorded. The criteria for inclusion in the study were an SIS score >1 and <3. Only one saline-treated animal fell outside this range (1.0) and was excluded. From this point on the study was blinded, ensuring that the researchers performing subsequent analyses (functional, morphological and transcriptomic) were not aware of which SCI treatment group the rats belonged to. Animal identities were decoded at the end of the data collection.

### Functional testing

A number of commonly used functional tests were conducted at 6 weeks post-injury (±0.5 weeks) by 2–3 independent observers. All observers were blinded to animal identity and treatment group during the testing. Open field locomotion and gait were assessed using the Basso, Beattie and Bresnahan (BBB) scale ( Basso *et al.*, 1995) as animals walked across a flat surface. Complex coordinated motor function was assessed by the animals’ ability to traverse a horizontal ladder of 76 cylindrical metal bars (3 mm thick, 7 mm apart) in which 15 bars had been removed (bars: 2, 6, 14, 22, 26, 33, 42, 44, 49, 54, 56, 62, 64, 66, 74 were selected using a random number generator; Haahr, 1998). Animals were pre-trained on the horizontal ladder prior to the commencement of the study. On the day of functional testing, the animals were allowed three practice runs prior to recording. Animals were then video recorded during three attempts at crossing the ladder and the footage analysed by a blinded observer to count the number of hind limb foot faults (foot slipping below the ladder between rungs). A tapered beam test was used to analyse complex coordinated motor function and balance. This test requires animals to walk along a narrowing beam suspended 1.2 m above the ground. The beam tapered from a width of 60 mm to 15 mm over a distance of 1.35 m. A slim ledge 1 cm below the beam on either side automatically caught and counted a foot fault each time one of the animal’s legs slipped off the beam and came into contact with the ledge. Animals were pre-trained on the beam prior to the commencement of the study. On the day of functional testing, the animals were allowed three practice runs along the beam prior to recording. This task was repeated six times and the average number of foot faults recorded. Each animal was video recorded when swimming in a tank of water (27–31°C) and the recordings analysed to determine if the animal used their hind limbs to swim, indicative of preservation of supra-spinal connections ( Magnuson et al., 2009; Saunders *et al.*, 1998; Smith *et al.*, 2006), and whether there were alternating hind limb movements.

### Histological analysis

At the end of each experimental period (24 h or 6 weeks post-injury) injured animals (together with age-matched controls) were terminally anaesthetized with an overdose of inhaled isoflurane (Lyppard Australia), the chest cavity opened and transcardially perfused with 50 ml heparinised (5 IU/ml) PBS (80 ml/min/kg) followed by 150 ml of 4% paraformaldehyde solution.

The spinal cords were dissected out and a 10 mm segment centred on and enclosing the injury site removed and post-fixed overnight in Bouin’s fixative (Sigma-Aldrich, Australia). Cords were dehydrated through increasing concentrations of ethanol then cleared overnight in chloroform before being mounted in paraffin wax (Paramat, VWR International). The embedded cord segments were serially sectioned in the transverse plane (Leica RM2125RT microtome, 5 μm thick sections) and sequential ribbons of 10 sections mounted on numbered glass slides. Slides were dewaxed, cleared through histolene (Fronine, Australia) and hydrated through decreasing concentrations of ethanol. Standard procedures were used for Hematoxylin and Eosin (H&E; Sigma-Aldrich) and Luxol Fast Blue (LFB; Gurr UK) staining. For immunohistochemical stains, peroxidase and protein blockers were applied (DAKO, 2 h each) before overnight incubation with primary antibodies (1:500 monoclonal mouse anti-CNPase, Sigma-Aldrich C5922 or 1:500 polyclonal rabbit anti-FOX3, abcam Ab104225) at 4°C. Secondary antibodies (1:100 polyclonal horse anti-mouse biotinylated, VECTOR BA2001 or 1:200 polyclonal swine anti-rabbit, DAKO Z0196) were administered and incubated for 1.5 h before application of either ABC kit (DAKO) or polyclonal rabbit PAP (Sigma-Aldrich P1291) complexes respectively. For both methods the final reaction product was developed with the DAB ^+^ kit (DAKO).

### Quantification of immunocytochemical staining

Tissue sections were viewed and photographed (with scale bars) using a light-microscope (Olympus BX50 fitted with a DP60 digital camera). Areas of positive stained tissue were measured using ImageJ ( Abràmoff *et al.*, 2004). The embedded scale bar in each image was used to calibrate the pixel-to-distance ratio, the perimeter of positive staining outlined manually and the enclosed area measured. In sections containing a central cystic cavity, the area of the cystic cavity was subtracted from the total cord cross-sectional area of the section to determine the area of remaining tissue.

**Figure 1.**
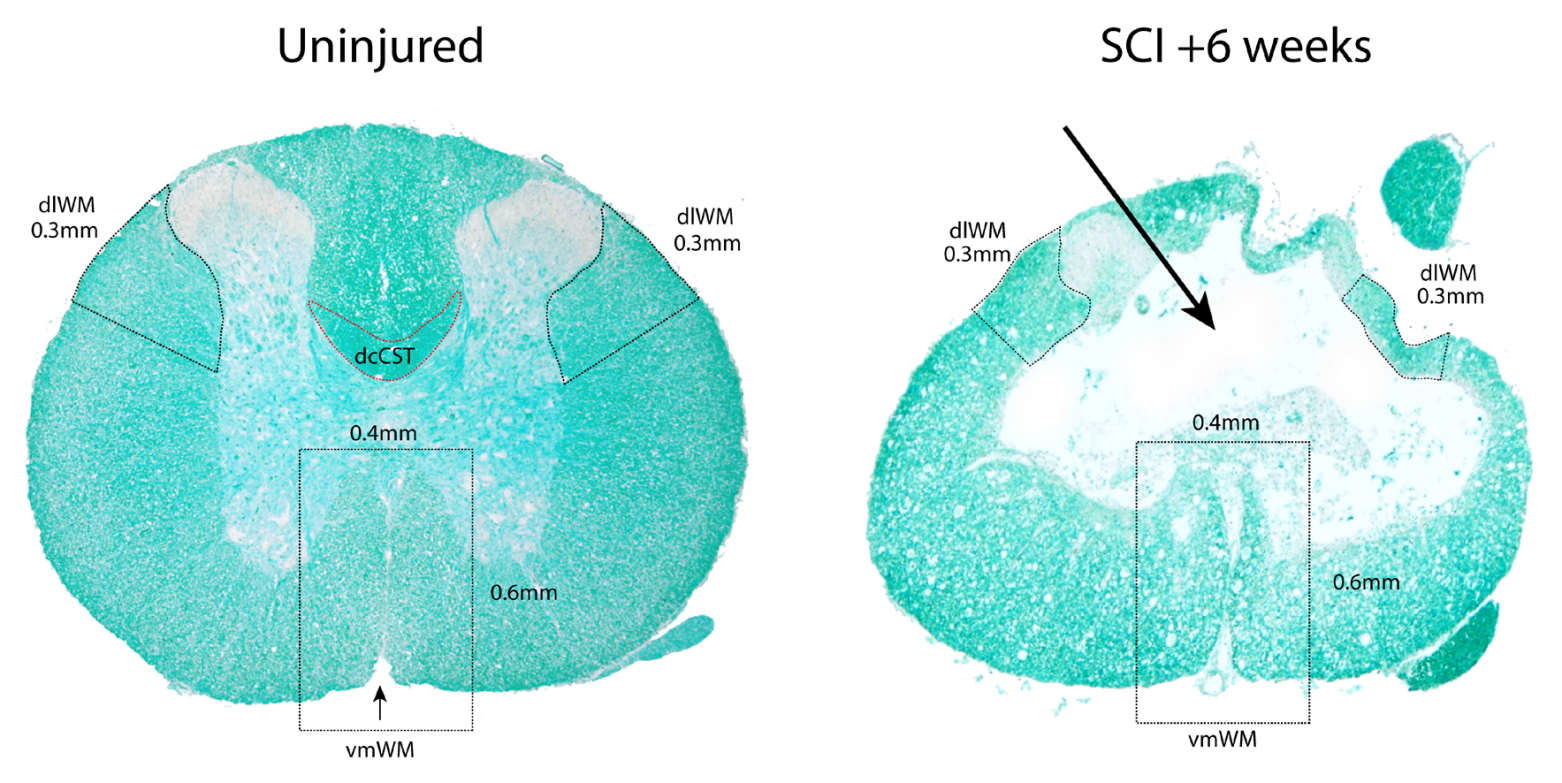
Transverse sections of thoracic (T10) level spinal cords stained with luxol fast blue (LFB). Left is from an uninjured control animal and right is from the injury centre in an animal 6 weeks after SCI. The outlined regions (black dashed lines) mark the areas used for measurements of dorsolateral and ventromedial white matter (dlWM and vmWM respectively). The red outline indicates the position of the dorsal column corticospinal tract (dcCST) at the base of the dorsal column. The dlWM regions were defined as all stained white matter from the ventral side of the dorsal horn down to 0.3 mm along the outer circumference of the cord. Both the left and right dlWM areas were measured. The vmWM was defined as the total area of stained tissue within a rectangle measuring 0.4 mm × 0.6 mm centred on the sulcal fissure (small black arrow in left image). The absence of most of the dorsal column and dcCST at 6 weeks post-injury is indicated by the large arrow in the right image.

Specific tissue regions containing some of the white matter tracts involved in hind limb motor function were outlined and measured separately. The dorsal column corticospinal tract (dcCST) located at the base of the dorsal column was not measured because it was no longer present at 6 weeks post-SCI. The rubrospinal tracts (RST) and dorsolateral corticospinal tracts (dlCST) are located in the dorsolateral white matter (dlWM) just below the dorsal horns. Preserved dlWM area was defined as positive stained tissue bounded by the lower edge of the dorsal horn at the dorsal extremity and a line drawn at right angles to the pial surface of the cord at a point located 0.3 mm down the outer circumference at the ventral extremity (shown in Figure 1). For each cord, the sum of the left and right dlWM preserved areas was recorded.

The ventro-medial white matter (vmWM) showed substantial tissue damage (“holes”) in all injured animals and thus simply outlining the perimeter of positive staining did not provide an accurate assessment of the true extent of tissue preservation. To estimate the actual area of positive tissue staining, a 0.4 mm and 0.6 mm rectangle was placed over the ventromedial region centred on and enclosing the sulcal fissure ( Figure 1). The number of pixels within the rectangle that were above a baseline threshold colour (the minimum colour considered to reflect positive staining) was recorded and the corresponding area calculated using the known pixel-to-distance ratio for the image ( *i.e*. the embedded scale bar). All dlWM and vmWM measurements were recorded as an average of two sections (5 to 10 sections/25–50 μm apart) from the centre of the injury site. The centre of the injury in each cord segment (section with the smallest preserved tissue area) was determined from serial reconstructions of tissue area in H&E sections equally spaced along the entire length of the cord segment. All LFB and CNPase dlWM and vmWM measurements were made within ± 450 μm of the lesion centre ( *i.e*. ± 9 slides).

To count the number of FOX3-positive grey matter neurons, images of the stained spinal sections were manually counted using the count tool in Photoshop (Adobe Systems). This places a sequentially numbered spot on top of each immunopositive cell. The image together with numbered spots is saved and compared with the same image counted by the other assessors.

### Statistical analyses

The functional performance of PcTx1-treated and saline-treated animals was compared using two-way analysis of covariance (ANCOVA, Prism v6, Graphpad, San Diego, USA). This method uses the General Linear Model approach and fits least squares linear regression lines to the raw data for individual animals then compares the slopes (correlations) of the regression lines. If the slopes are found to be equal, the intercepts (elevations) are then compared. Differences in the initial injury severity between animals within each group introduces a covariate that makes a substantial contribution to the total observed variance in each treatment group. The presence of this covariate negates the use of simple parametric testing such as ANOVA and Student’s *t*-Test.

### Blood-spinal cord barrier integrity

Blood-spinal cord barrier (BSCB) function at the lesion site was assessed between 2 h and 7 days post-SCI for different size permeability tracers ( *n* = 2 per tracer and time point) in a separate series of 38 untreated, injured rats. At 30 minutes prior to the time of BSCB assessment, animals were anaesthetised with urethane (1–1.5 g/kg, i.p.) and a femoral vein injection (100 μl) containing one of the following permeability tracers dissolved in sterile saline was administered: 286 Da biotin ethylene diamine (BED, A-1593 Life Technologies Australia), 3 kDa biotin-dextran-amine (BDA, D3308, Life Technologies Australia), 10 kDa dextran rhodamine B (DRB, D-1824, Life Technologies Australia), 44kDa horseradish peroxidase (HRP, P-8375 Sigma-Aldrich, Australia) or 70 kDa dextran fluorescein (DF-70kDa, A-1823, Life Technologies Australia). After 20 minutes of circulation time the animals were administered an overdose of anaesthetic (inhaled isoflurane). A 10 mm segment of spinal cord enclosing the injury site was removed post-mortem and immersion-fixed in 4% paraformaldehyde. For animals injected with HRP or BED, the cord segments were embedded in 4% agar, serially sectioned (70 μm thickness) in a vibrating microtome (Leica VT1000S) and stained with DAB (D-5905, Sigma-Aldrich, Australia). For animals injected with the dextran tracers, the spinal segments were embedded in paraffin wax, serially sectioned in the transverse plane at 5 μm thickness and mounted on glass slides (10 sequential sections per slide). Sections approximately 0.5 mm apart along the entire length of the cord segment were inspected under fluorescence microscopy. BSCB function was interpreted as disrupted where tracer was visible outside blood vessels within the spinal tissue and interpreted as intact where the tracer was completely confined to the lumen of blood vessels.

### Transcriptomics: RNASeq (Illumina platform)

PcTx1 ( *n*=3) and saline treated animals ( *n*=3) were sacrificed 24 h after injury (overdose of inhaled isoflurane), the thoracic region of the spinal cord was removed under RNase-free conditions and 3 mm long segments collected from the injury centre, the adjacent cord rostral to the injury centre and the adjacent cord caudal to the injury centre. Each 3 mm segment was placed into separate cryovials containing RNA-later solution (Life Technologies Australia) for 2 h before being snap frozen by placing the cryovials into liquid nitrogen. Total mRNA in each sample was extracted using commercial kits (RNeasy, Qiagen) and stored at -80°C. Transcriptome datasets were generated using RNAseq analysis (Illumina HiSeq 2000 platform, 100 bp single end reads, Australian Genome Research Facility, AGRF, Melbourne, Australia). Analysis of differential transcript expression between PcTx1-treated and saline-treated animals was conducted using edgeR software with sample weights ( Robinson *et al.*, 2010; Saunders *et al.*, 2014; Zhou *et al.*, 2014). The significance level was set as greater than 2 fold-change (FC) in the positive or negative direction.

Analysis of transcript variance revealed effects of initial lesion severity (SIS scores) between individual animals within each treatment group (see Transcriptomic analysis below). Accordingly, subsequent analysis was made comparing only datasets from animals with similar sized initial injury severities.

FC values were calculated from FKPM (fragments per kilobase of transcript per million mapped reads) values in the PcTx1-treated vs saline-treated groups for animals with similar SIS scores. Positive fold changes indicate that the genetic transcript was present in a higher proportion in PcTx1-treated animals in that part of the cord compared to saline-treated animals, with negative values indicating lower proportions in PcTx1-treated animals. Gene set testing was conducted to identify enriched pathways using the Gene ontology ‘Panther’ classification system ( Mi *et al.*, 2013).

## Results

### Assessment of the severity of the initial injury

Despite the fact that all animals received the same mechanical impact to the spinal cord (2 mm diameter tip, 0.30 m/s ± 0.04 S.D. impact velocity, 1.44 mm ± 0.01 S.D. penetration depth, 0.997s ± 0.004 S.D. compression time), the resulting initial injury severity (SIS) scores ranged from 1.50 to 2.75 ( Table 1). This reflects the inherent variability of spinal tissue injuries even when conducted using a well-validated impactor ( Ek *et al.*, 2010) operated by an experienced experimenter. There was no correlation between any of the impact parameters and SIS scores (data not shown). There was also no significant difference between the average SIS scores of the PcTx1-treated and saline-treated groups ( Table 1). However, within each treatment group there was a range of initial injury severities and for this reason each animal’s results were plotted against their initial SIS score for analyses.

**Table 1.** Comparisons of mean initial injury severity (SIS scores) in the PcTx1-treated and saline-treated groups of animals (assessments made during the first 24 h post-SCI). All animals received the same mechanical impact to the exposed lower thoracic (T10) spinal cord.

Initial Injury Severity Scores		Saline-treated	PcTx1-treated	ANOVA (oneway)
SCI + 24 hours	mean ± s.d.	2.11 ± 0.50	2.31 ± 0.40	p = 0.44
	range	1.50 – 2.75	1.75 – 2.75	
	n	7	6	
SCI + 6 weeks	mean ± s.d.	2.00 ± 0.44	2.23 ± 0.37	p = 0.31
	range	1.50 – 2.75	1.75 – 2.75	
	n	10	6	

**Table 2.** A simplified injury severity (SIS) scale based on simple observable criteria for assessing initial gross locomotor functional deficits soon after spinal cord injury in rats. Animals are first assessed by the presence (scores 0–1.5) or absence (scores 2–3) of weight supported hind limb stepping. Those exhibiting weight support are then graded on the basis of the visual severity of any gait abnormality (none, slight or gross). Those without weight support are graded on the basis of how many joints (hip, knee, ankle) they are able to voluntarily flex/extend (none, one or more than 1) when restrained by holding the tail and lifting the hind quarters gently up and lowering back down. Each hindlimb is assessed independently and the average of both recorded. The most equivalent grades on the scale used by Herrmann *et al.* (2008) and Anderson *et al.* (2016) are shown on the right.

Simplified Injury Severity (SIS) Grading Scale					
Weight support	Stepping pattern	Extend & flex joints	SIS Score	Equivalent Herrmann Grading	
YES	Normal	all	**0**	hindlimb function essentially normal	**5**
	Slight abnormality	all	**1**	hindlimb used for weight support and stepping, but obvious disability	**4**
	Gross abnormality	all	**1.5**	hindlimb assisted in occasional weight support and plantar placement, but not in stepping	**3**
NO	No stepping	two or more	**2**	hindlimb movements obvious, but did not assist in weight support or stepping	**2**
		one	**2.5**	little voluntary hindlimb movement	**1**
		none	**3**	no voluntary hindlimb movement	**0**

### Functional performance at 6 weeks post-injury

**Figure 2.**
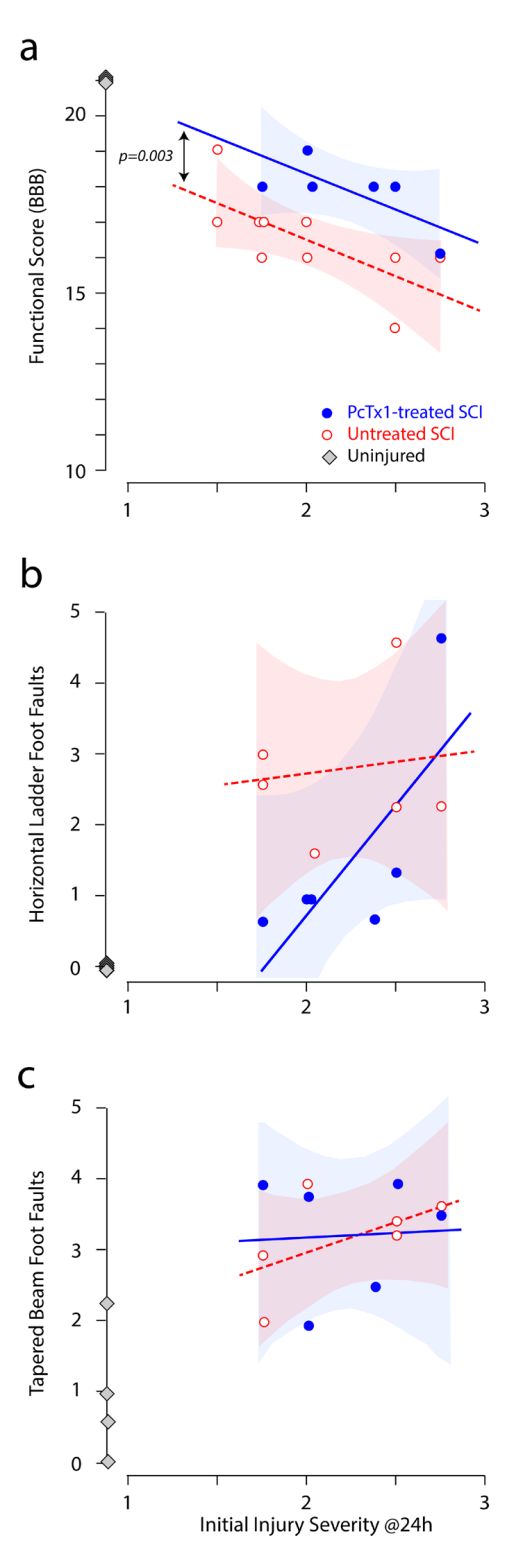
Effect of PcTx1 treatment on hind limb function at 6 weeks after SCI. Locomotor functional data are plotted against the initial injury severity (SIS scores); ( **a**) BBB functional scores, ( **b**) horizontal ladder and ( **c**) tapered beam. Each data point represents an individual animal, PcTx1-treated (blue circles), saline-treated (red open circles) and uninjured controls (grey diamonds). Lines in ( **a**) were fitted by least squares linear regression. The datasets in ( **a**) were best described by two separate lines with the same slope (-1.9681), but with a statistically significant difference in elevation (F _1,9_=16.1324, p=0.003, ANCOVA). The datasets in ( **b**) and ( **c**) were not significantly different. The shaded areas indicate the 95% confidence intervals around each regression line.

At 6 weeks post-injury, there was an inverse correlation between initial injury severity (SIS) scores and residual hind limb function (BBB gait scores, Figure 2a). Over the entire range of initial injury severities, PcTx1-treated animals scored higher on the BBB scale than saline-treated rats. Linear regression and ANCOVA revealed that the data were best described by two separate lines having the same slope, but with the PcTx1-treated group having a significantly higher elevation compared to the saline-treated group (F _1,9_=16.1324, p=0.003). Uninjured control rats (diamond symbols in Figure 2a, *n*=4) all scored 21 on the BBB scale.

Figure 2b shows the correlation between the initial SIS scores for individual rats and their average number of hind limb food faults on the horizontal ladder task (average from three repeat attempts). For a given SIS score, PcTx1-treated rats tended to make fewer hind limb errors than saline-treated animals ( *n*=6 per group). No significant correlation was observed between SIS scores and foot faults. Control rats ( *n*=4) made no hind limb errors along the ladder. There was no significant difference between the two treatment groups on the tapered beam task ( Figure 2c) and no correlation between initial SIS scores and average foot faults (from six attempts) on the tapered beam (not shown).

In the swimming test, all PcTx1- and saline-treated rats were able to kick their hind limbs in a coordinated manner indicating the presence of some supraspinal control of hind limb function. There was no observable differences between the two treatment groups in the pattern and style of swimming.

### Preservation of individual white matter tracts at 6 weeks post-injury

Analysis of FOX3 immunopositive neurons, total cross-sectional tissue area (as shown by H&E staining) or general cross-sectional white matter area (as shown by CNPase and LFB staining) did not show any significant differences between PcTx1-treated and saline-treated rats at any point along the spinal cord at 6 weeks post-injury. Thus an in-depth analysis of regions of locomotor importance at the injury centre was conducted.

**Figure 3.**
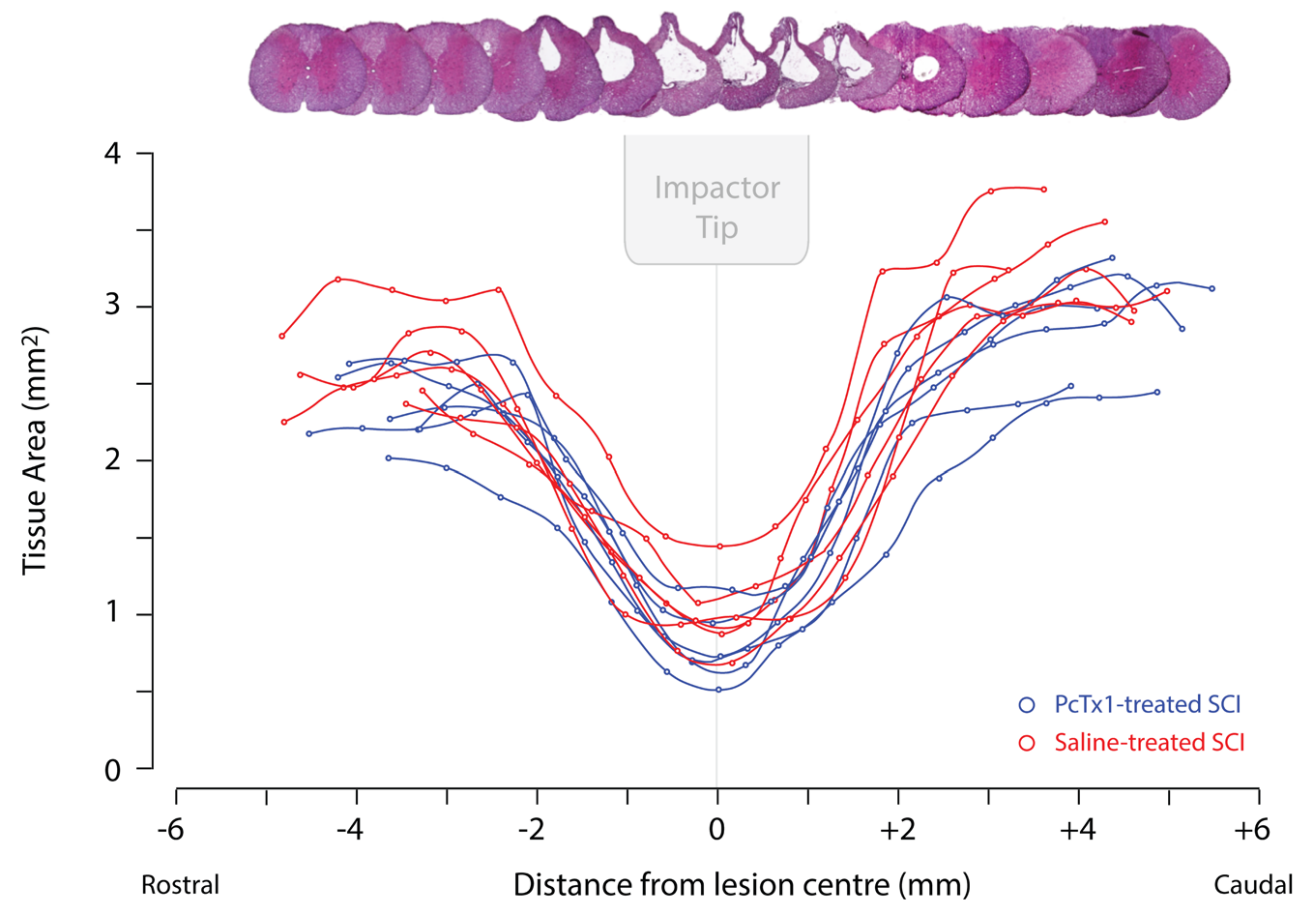
Cross-sectional areas of tissue in serial H&E stained transverse sections along the length of the spinal cord at 6 weeks post-injury. Areas of preserved tissue were calculated as the total cord area minus the area of the central cystic cavity. PcTx1-treated rats (blue lines; *n*=6) and saline-treated rats (red lines; *n*=6) are shown. Negative values on the x-axis indicate regions rostral to the injury centre (0) and positive values indicate regions caudal to the injury centre. A visual reconstruction of serial H&E traverse sections corresponding to equivalent points along the graph is shown. The relative size and position of the impactor tip at the time of injury is also indicated.

**Figure 4.**
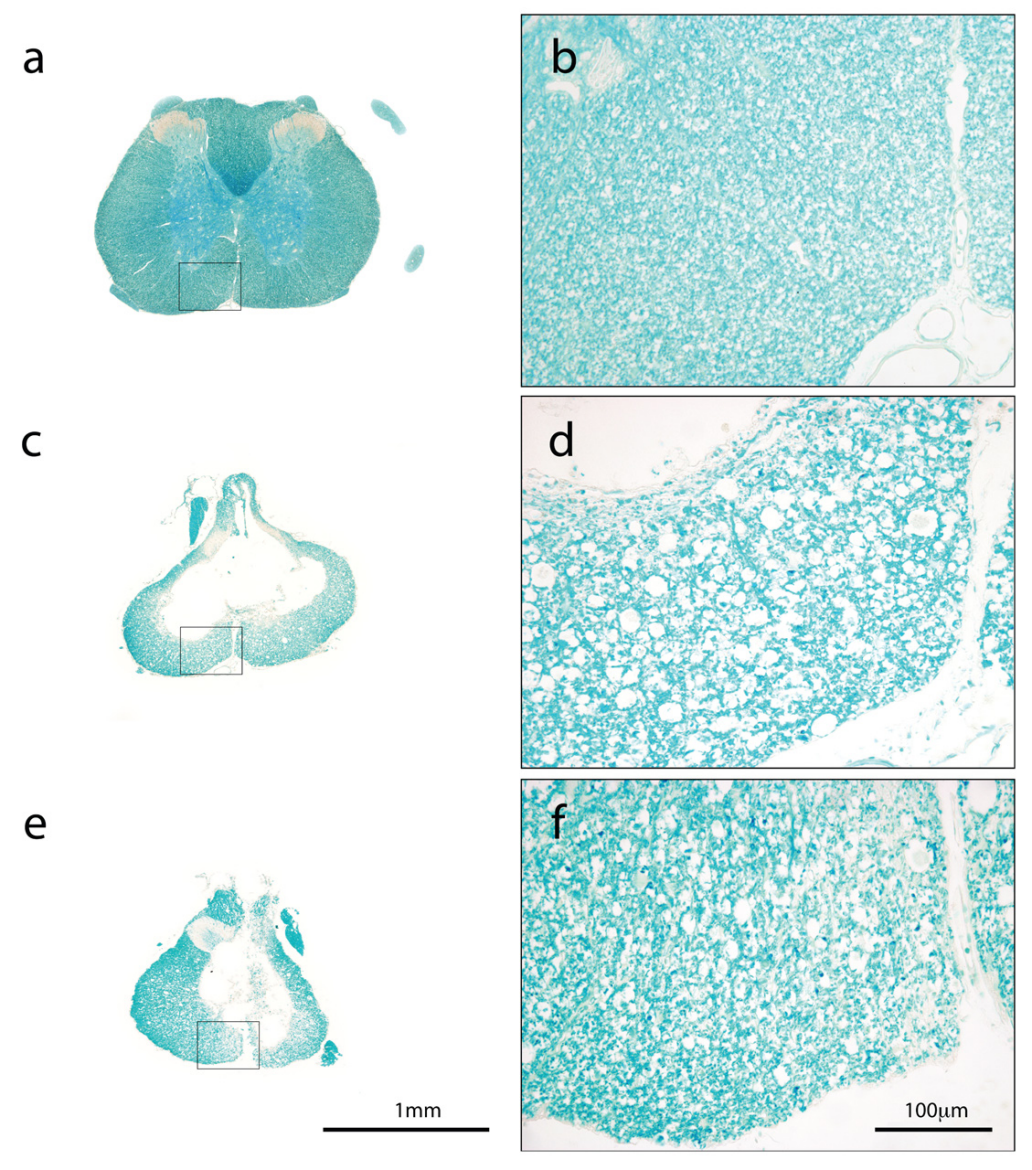
Luxol fast blue (LFB) stained transverse cross-sections from the injury site of an uninjured control ( **a** and **b**), saline-treated ( **c** and **d**) and PcTx1-treated ( **e** and **f**) animals. The outlined areas in ( **a**), ( **c**) and ( **e**) are shown at higher magnification in ( **b**), ( **d**) and ( **f**) to illustrate the preservation of myelin in the ventromedial areas of these cords. Note the increased numbers and sizes of myelin-devoid spaces in the saline-treated cord ( **d**) compared to the PcTx1-treated cord ( **f**). The saline-treated rat ( **c** and **d**) had an initial simplified injury severity score (SIS) of 1.73 and the PcTx1-treated rat ( **e** and **f**) had an SIS of 1.9.

For a white matter tract in the spinal cord to be functional it requires an uninterrupted connection between the brain nuclei from which the tract axons originate and synaptic connections further down the spinal cord. Thus only the tracts that run all the way through the injury centre would have functional significance for hind limb locomotion. The injury centre was determined by plotting the H&E stained area along the cord ( Figure 3) and selecting the point of the least remaining tissue. In the rodent spinal cord, supraspinal motor fibres descend in a number of areas of the cord. The main corticospinal tracts run in the dorsal column (dcCST) and the dorsolateral white matter (dlCST) ( Steward *et al.*, 2004). Rubrospinal and reticulospinal tracts are also thought to be involved in hind limb locomotion ( Watson & Harrison, 2012). In this study, the dcCST was completely destroyed and no longer present at 6 weeks post-injury in all rats (See Figure 1). The area of LFB staining in the ventromedial white matter (vmWM) at the injury centre was highly variable and showed no difference between treatment groups. There was, however, a noticeable difference in the appearance of myelin in this region between treatment groups as illustrated in Figure 4.

**Figure 5.**
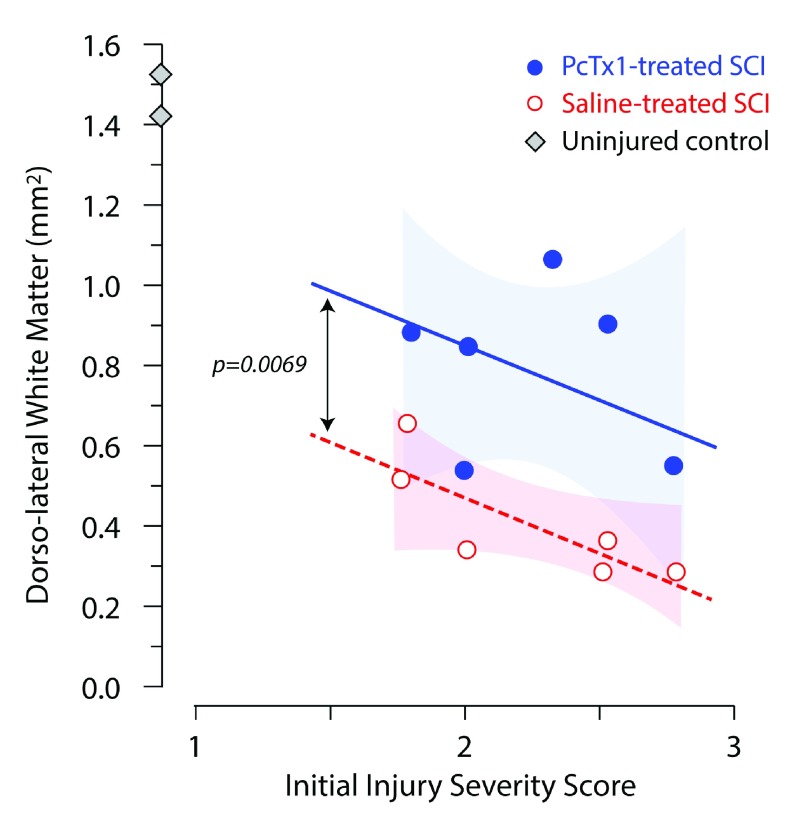
Cross-sectional areas of luxol fast blue (LFB) staining of the dorsolateral white matter (dlWM) 6 weeks after injury plotted against each rats’ initial injury severity (SIS score). Saline-treated rats (red open circles; *n*=6), PcTx1-treated rats (blue circles; *n*=6) and control rats (grey diamonds; *n*=2) are shown. All measurements were the average of two sections from the injury centre of each animal. The lines shown were fitted by least squares linear regression. The data were best described by two separate lines with the same slope (-0.0237), but with a statistically significant difference in elevation (F _1,8_=12.9908, p=0.0069, ANCOVA). The shaded areas indicate the 95% confidence intervals around each regression line.

In PcTx1-treated animals, myelin showed fewer axotomised tracts and a denser staining pattern compared to saline-treated SCI controls ( Figure 4). In addition, PcTx1-treated animals had significantly larger areas of preserved dorsolateral white matter (dlWM) compared to saline-treated animals ( Figure 5). ANCOVA revealed two separate lines with the same slope best described the data with the PcTx1-treated group having a significantly higher elevation ( Figure 5; F _1,8_=12.9908, p=0.0069). Similar results were obtained from adjacent sections stained with H&E or CNPase (data not shown).

**Figure 6.**
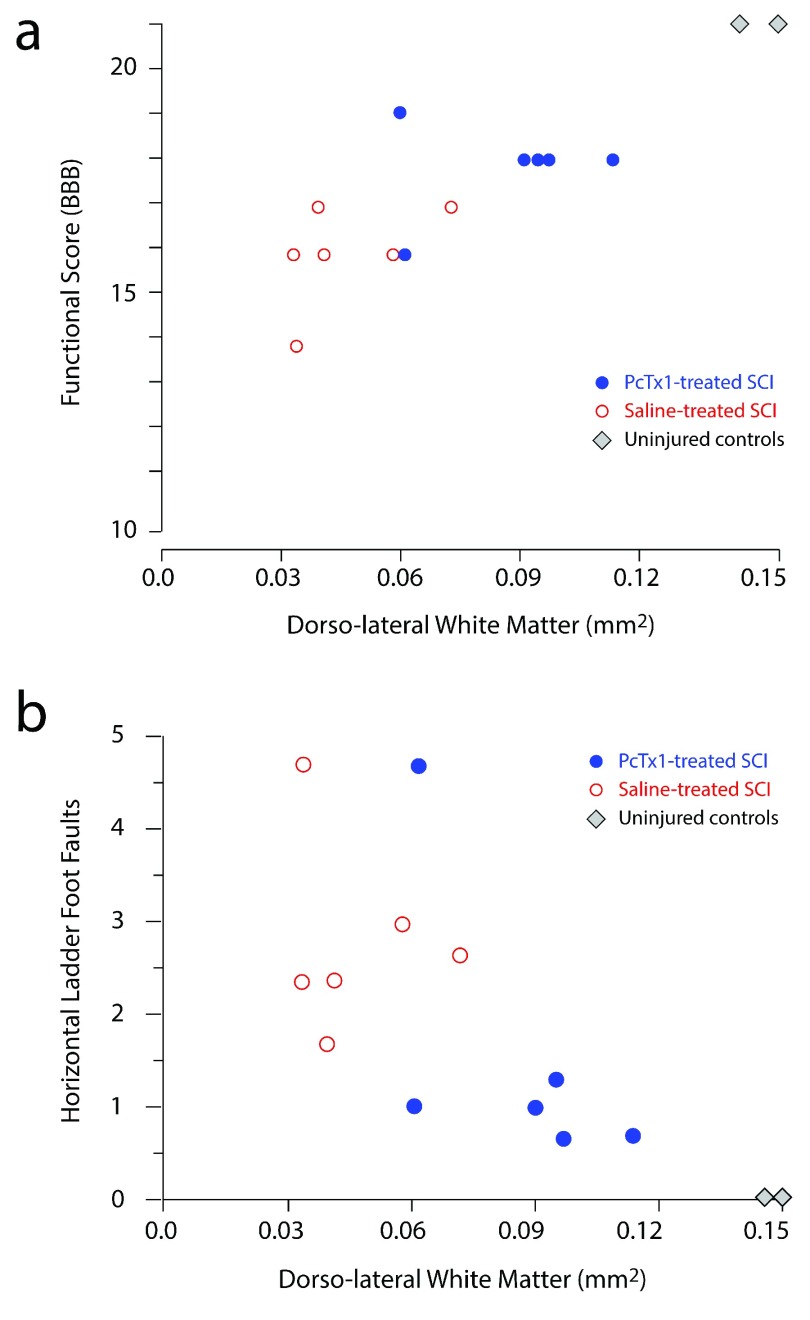
Correlations between functional scores and area of luxol fast blue (LFB) staining in the dorsolateral white matter (dlWM) regions. Cross-sectional areas of LFB staining in each animal at 6 weeks post-injury were measured as the average of two sections from the centre of the injury. ( **a**) BBB gait analysis ( **b**) horizontal ladder foot faults. Each data point represents an individual animal, PcTx1-treated rats (blue circles; *n*=6) and saline-treated rats (red open markers; n=6).

A positive correlation was observed between the preserved area of LFB staining in the dlWM at the injury centre and functional performance assessments using BBB scores ( Figure 6a) and the horizontal ladder task ( Figure 6b). There was no correlation between LFB area in the dlWM and performance on the beam task (not illustrated). PcTx1-treated rats typically had larger areas of preserved dlWM that correlated with better functional outcomes.

### Morphology at 24 h post-injury

Although 6 weeks post-injury is a suitable time-point for long-term functional assessment, it is well after the response to injury has concluded. Thus, analysis was also conducted at an earlier 24 h post-injury time-point, when white matter loss is still continuing ( Ek *et al.*, 2010; Ek *et al.*, 2012), to determine the potential mechanisms of PcTx1 neuroprotection.

Assessments of tissue area using H&E and LFB staining could not be accurately performed at this time due to the presence of significant amounts of blood within the cord and the distinction between live and recently dead tissue is not apparent, both of which affect interpretation of staining. The only morphological analyses that were possible to meaningfully conduct were immunocytochemistry for FOX3 and CNPase. No significant differences were observed between PcTx1-treated and saline-treated groups at 24 h post-injury in terms of the average numbers of FOX3 immunopositive neurons at any point along the 10 mm length of cord segment (not illustrated).

Total white matter area as determined by measurements of CNPase positive staining revealed preservation of tissue at the injury centre in PcTx1-treated animals with higher severity injuries (SIS ~2.5, 1.27 mm ^2^ and 1.05 mm ^2^ in PcTx1-treated versus 0.631 mm ^2^ and 0.734 mm ^2^ in saline-treated), but not in animals with lower severity injuries (SIS 1.5, 1.38 mm ^2^ in PcTx1-treated versus 1.40 mm ^2^ in saline-treated). This greater effect of the PcTx1 treatment in animals with more severe injuries was also apparent in the dlWM area measurements (SIS ~2.5, 0.166 mm ^2^ and 0.137 mm ^2^ in PcTx1-treated versus 0.09 mm ^2^ and 0.056 mm ^2^ in saline-treated compared to SIS 1.5, 0.143 mm ^2^ in PcTx1-treated versus 0.147 mm ^2^ in saline-treated). This suggests that PcTx1 treatment was more effective at preserving white matter in animals with more severe injuries at 24 h post-SCI.

### Transcriptomic analysis

RNAseq transcriptomic analysis of the spinal cord at 24 h after a contusion injury was performed on PcTx1-treated and saline-treated animals ( *n*=3 per group) using the Illumina Platform. The SIS scores in the PcTx1-treated animals were: 1.9, 2.5 and 2.75 while in the saline-treated group they were: 2.5, 2.5 and 2.75. Initial differential analysis of the datasets compared the means ( *n*=3) of the PcTx1-treated and saline-treated groups. This highlighted a large number of genes that were significantly differentially expressed (FC>2 or FC<-2): 516 genes rostral to the injury site, 136 genes at the injury centre and 133 genes caudal to the injury site.

**Figure 7.**
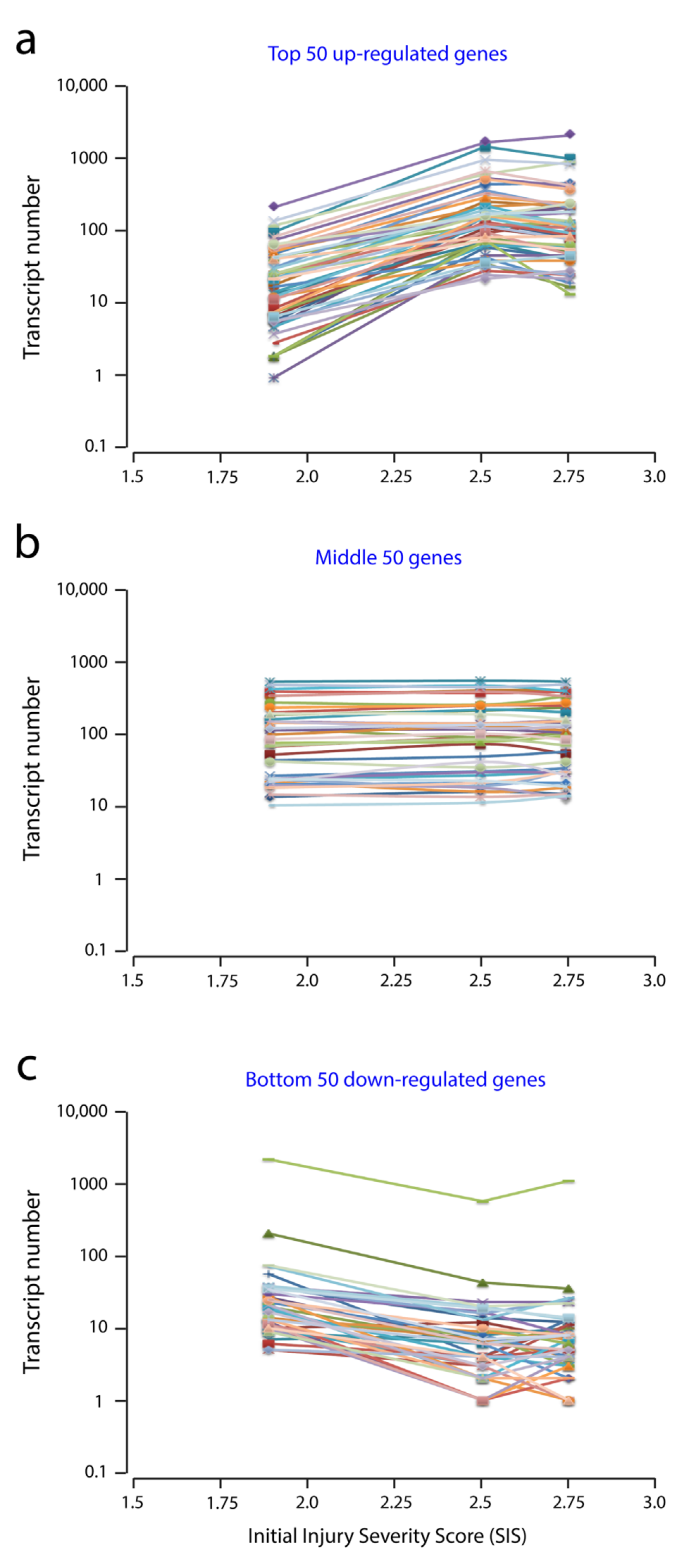
Correlation between raw transcript numbers and initial injury severities (SIS scores) in the rostral segment of spinal cords from PcTx1-treated rats at 24 h post-injury (Illumina Platform). ( **a**) shows the top 50 up-regulated genes, ( **b**) the middle 50 unchanged genes and ( **c**) the bottom 50 down-regulated genes ( Table 3). Each line represents a single gene. Data shown are for the rostral segment, similar correlations were observed in the injury and caudal segments. Note, transcript numbers increased ( **a**) or decreased ( **c**) for each gene with increasing injury severity (SIS) score. This highlights the importance of using similar size initial injuries for comparative studies.

**Figure 8.**
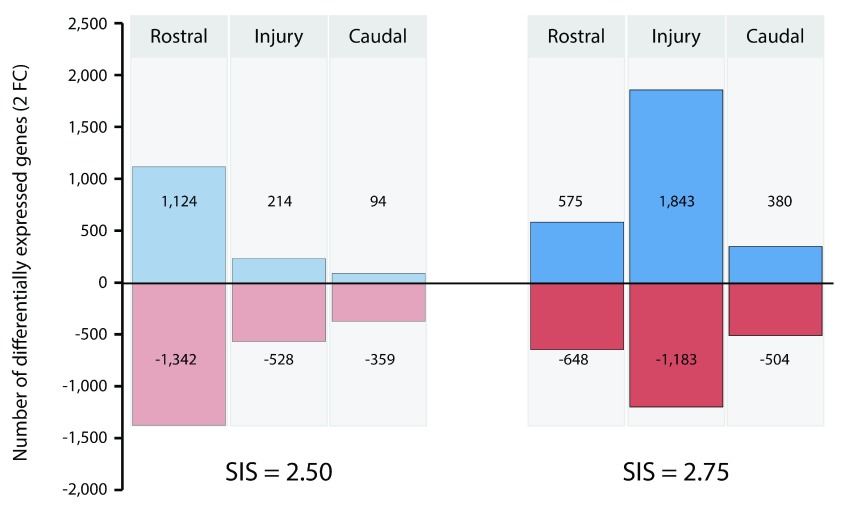
Comparison of the numbers of transcripts in the rostral, injury and caudal segments that changed (FC>2 or FC<-2) between PcTx1-treated and saline-treated animals. Datasets obtained from animals with 2.5 and 2.75 SIS scores. Negative values (red) indicate the number of down-regulation genes while positive values (blue) indicate the number of up-regulated genes.

However, marked inter-animal variance was apparent in the transcript numbers of many of the differentially expressed genes. When transcript counts for the top 50 up- and down-regulated genes were plotted against the initial injury severity (SIS score) for each rat, it was apparent that the severity of the initial injury had a profound effect on gene expression levels (see Figure 7). Accordingly, transcript fold changes were calculated between animals with similar sized injuries. Figure 8 shows gene expression changes between PcTx1-treated and saline-treated animals with 2.5 and 2.75 size initial injury severities. Not only was there a difference in the number of up-regulated and down-regulated genes between the two injury sizes, but there were also marked differences in the panels of differentially expressed genes in different segments of the spinal cord. Table 3 lists the top 50 genes identified as being significantly up- or down-regulated by PcTx1 treatment for each spinal cord segment in animals with SIS scores of 2.5 ( Table 3a) and 2.75 ( Table 3b). Datasets for all genes with FC >2 or <-2 can be found in Tables S1 (see Supplementary file 1). As seen in Table 3a (SIS 2.5) the magnitude of highly up-regulated genes (FC>20) was highest in the rostral (10 genes) and injury segments (5 genes), whilst in the caudal segment no gene was up-regulated by more than FC 7.5, with most being less than FC 5. The largest FC in the down-regulated dataset, however, was identified in the caudal segment (FC -35.3). The three segments showed very little, if any, overlap in the top 50 up- or down-regulated genes ( Tables 3a & 3b).

**Table 3.** Top 50 genes that at 24 h after SCI were either up-regulated or down-regulated in response to PcTx1 treatment. Data are shown for the injury centre segment and the immediately adjacent rostral and caudal segments for animals with SIS 2.5 injuries (a) or SIS 2.75 injuries (b). Fold changes (FC) were calculated from FPKM values (see Methods).

SIS 2.75											
Rostral				Injury Centre				Caudal			
Up-regulated		Down-regulated		Up-regulated		Down-regulated		Up-regulated		Down-regulated	
Gene	FC	Gene	FC	Gene	FC	Gene	FC	Gene	FC	Gene	FC
Cxcl2	20.2	Micb	-18.5	Slc17a7	41.1	Myh1	-29.8	Scn10a	74	Mmp10	-39.7
Dnah8	18.4	Slc19a3	-12	Kcnh7	27.7	Actn3	-29.3	Ckm	73	Serpinb2	-32.2
Rn60_X_0663.2	16.2	Slco1c1	-11.6	Ksr2	26.8	Ereg	-27.1	Mylpf	42.3	LOC100911361	-32.2
Gabrp	16.2	Gm26965	-11.1	Skor1	26.8	Hp	-26	Avil	28.6	Acap1	-21.8
Il31ra	11.9	Npas1	-10.2	Frmpd3	25.8	Treml2	-21.7	Car3	22.9	Fst	-18.9
LOC100361444	9.7	Slc44a3	-10.2	Lbx1	22.1	Tnnt3	-19.5	Tnnt1	16.9	Siglech	-18
Stfa2l3	8.6	Siglec8	-10.2	Opn4	17.5	Arl5c	-18.4	Scn11a	16.9	Ccl12	-17.4
Il2ra	8.6	Kcns2	-9.7	Grin2a	16.8	Ccdc69	-16.3	Myh1	16.1	RGD1309870	-16.1
Cxcl3	8.3	Cxcl17	-9.3	RGD1308544	16.6	Acap1	-16.3	Actn3	13.7	AABR07030791.1	-15.1
Il1rl2	8.1	Kcnh6	-8.8	Rasal1	14.8	Itga2	-16.3	Gabrq	13.7	Il11	-13.6
Tfap2a	7.9	Akr1c19	-7.4	Frem3	14.8	Tbx15	-16.3	Kcnh8	12.7	Rpl17	-13.4
Twist2	7.9	Tph2	-7.4	Lypd6b	14.1	Il4i1	-16.3	Sprn	10.6	Fcrla	-13.2
Arl5c	7.6	Tctex1d1	-7.4	AABR07065531.5	13.8	RNaseP_nuc	-15.6	Acta1	10.2	Cxcl6	-12.8
AABR07053509.2	7.6	Ccdc13	-7.1	Slc26a8	13.8	Il11	-14.6	Tyrp1	9.5	Slc39a12	-11.2
Clca4l	7.5	Rpl30	-7.1	Nmu	13.8	AABR07021998.1	-14.1	AABR07070246.1	9	Trib3	-11
Fam111a	7.4	Fam183b	-6.9	Lgr5	13.8	Irg1	-13.5	RGD1560672	9	Gabrp	-10.4
Cyp4f18	7	Hpx	-6.8	Slc27a2	13.5	RNase_MRP	-13.1	Myl1	8.7	LOC100909752	-10.4
Dusp5	7	AABR07000733.1	-6.6	Hapln1	13.5	Irf4	-13	Siglec8	8.6	Gdf15	-10.4
Lrrc15	6.7	RGD1306233	-6.5	RGD1560672	12.9	Cytl1	-13	Tusc5	8.3	Dnah8	-9.5
Nppb	6.5	Hapln1	-5.8	Crhbp	12.9	Hcst	-12.6	Isl2	7.9	Cxcl3	-9.2
Gdnf	6.5	Kcng1	-5.7	Shisa6	12.3	Cxcl2	-12	Abca6	7.4	Serpine1	-8.6
Cyp27b1	6.5	Gm1043	-5.6	AABR07045621.1	12	LOC684828	-11.9	LOC100359656	6.9	Hsf2bp	-8.5
Oas3	6.5	Plin4	-5.6	Mfi2	12	Gdnf	-11.6	Zap70	6.7	Fam83g	-8.5
Ucn2	6.4	Chrm4	-5.2	AABR07070246.1	12	Tmprss11f	-11.6	Ifit1	6.3	Sp5	-8.5
AC091336.1	5.9	Creg2	-5.2	Cfap126	12	Myct1	-10.8	Slc26a3	6.3	AABR07044837.2	-7.9
Snai2	5.9	Chrm4	-5.2	Gpr61	11.8	Pf4	-10.8	Gprin3	5.8	Ccl17	-5.7
Igj	5.8	Tecta	-5.2	Myadml2	11.5	RGD1565355	-10.7	Zfp773	5.3	Oip5	-5.7
Hsh2d	5.8	AABR07034833.1	-5.1	Gbx1	11.1	Nppb	-10.3	Mfi2	5.3	Irg1	-5.7
Trh	5.7	Dnah5	-5.1	Fbxl18	11.1	Dusp2	-10	Esrrb	5	Ppp1r32	-5.7
Il11	5.4	Draxin	-5.1	Fstl4	11.1	Slpi	-9.9	Clic6	5	Cd7	-5.7
Slamf6	5.4	Gpr101	-5	Htr4	11.1	Selp	-9.8	Ctxn3	5	Ccl2	-5.8
Msc	5.4	Enkur	-4.9	Slc19a3	11.1	Krt12	-9.8	Dpf3	4.9	Mis18a	-5.9
Echdc3	5.4	RGD1304810	-4.9	Ky	11.1	Galnt5	-9.8	Icam5	4.8	Dusp2	-6
Rn60_7_1164.1	5.1	Myl1	-4.6	Pip5kl1	11.1	Trem3	-9.5	Ibsp	4.8	Fam111a	-6
Lif	5.1	Kcp	-4.6	Pax2	10.8	Fcnb	-9.5	Lypd8	4.8	Ucn2	-6.2
Osm	5.1	Rn60_X_0744.3	-4.6	Vsx2	10.8	RGD1305807	-9.2	Nox4	4.8	Phldb3	-6.2
Osr2	5	Pgr	-4.6	Gjd2	10.7	Col28a1	-9.2	Itih2	4.8	Btc	-6.2
Nox4	5	Dok7	-4.6	Dok7	10.6	Retnlg	-8.9	LOC299282	4.7	Pfkfb4	-6.3
Irg1	5	Opn4	-4.6	Efna5	10.6	Osm	-8.8	4932411E22Rik	4.6	Mar-01	-6.6
Rhbg	5	Mybpc1	-4.6	Tctex1d1	10.2	Trh	-8.8	P2rx3	4.6	Itga2	-6.6
Il1r2	4.9	Chodl	-4.5	Ppp1r3g	10.2	Dsc2	-8.7	Slc22a2	4.3	Rpl12	-6.6
AABR07001634.1	4.9	Cdh12	-4.5	Cxcl17	10.2	Cyp4f18	-8.7	Mybpc1	4.2	Gdnf	-6.6
Mmp8	4.8	RGD1308544	-4.4	Wnt3	10.2	Il17re	-8.7	Gabre	4.2	Lif	-6.6
LOC24906	4.8	Gdpd2	-4.4	Neurod6	10.2	Il1rl2	-8.7	RNase_MRP	4.2	Clca4l	-6.8
Myod1	4.6	Akr1b7	-4.4	Hecw1	10	Fam60a	-8.7	B3gntl1	4.2	Fabp4	-6.9
Rufy4	4.5	LOC498435	-4.3	Cpne9	10	Gfra3	-8.7	Btbd17	4.2	Eif5b	-7
Myo18b	4.5	Perp	-4.3	RGD1564053	9.7	Ucn2	-8.5	Glb1l2	4.2	Mmp3	-7
Pglyrp1	4.5	Spag1	-4.3	Col25a1	9.7	Fabp4	-8.5	Tnfsf10	4.1	Ereg	-7.6
Fabp4	4.5	RGD1564053	-4.2	Cntn6	9.6	Ccr7	-8.3	AC141169.2	4.1	Lamb3	-7.6
AC115145.1	4.4	Cntnap5a	-4.2	Abcc8	9.6	Kazald1	-8.3	Oprk1	4.1	Ccl7	-7.7

In contrast, a different pattern of expression was observed in animals with more severe injuries (SIS score of 2.75, Table 3b). In the rostral segment only one transcript was up-regulated by FC>20 and none were down-regulated by more than FC<-20. However, in both the injury and caudal segments there were several transcripts that were over 20 FC (both up- and down-regulated). The largest FC values were obtained for the caudal segment: two transcripts were up-regulated by FC>70 while three were down-regulated by FC<-30 ( Table 3b). Again, there was little, if any, overlap in the top 50 up- or down-regulated genes between the three segments.

The top 50 up-regulated and down-regulated genes for each segment were analysed by ‘String’ which orders them into functional protein association networks ( Szklarczyk *et al.*, 2015). These are illustrated in Supplementary file 2. A common feature of the protein association pathways was chemokine signalling (and immune response) related proteins. In both the SIS 2.5 and SIS 2.75 groups, PcTx1-treated rats showed up-regulation of chemokine-based signalling in the rostral segment and a down-regulation in the caudal segments. In the injury segment of the spinal cord initial injury severity appeared to be the key contributor to chemokine signalling – with a cluster of genes up-regulated in the SIS 2.5 group and a cluster down-regulated in the SIS 2.75 group. Along with chemokine-related proteins, smooth muscle associated proteins were up-regulated in the injury centre of the SIS 2.5 group. These results suggest PcTx1 may act through modifying the inflammatory response to SCI.

**Figure 9.**
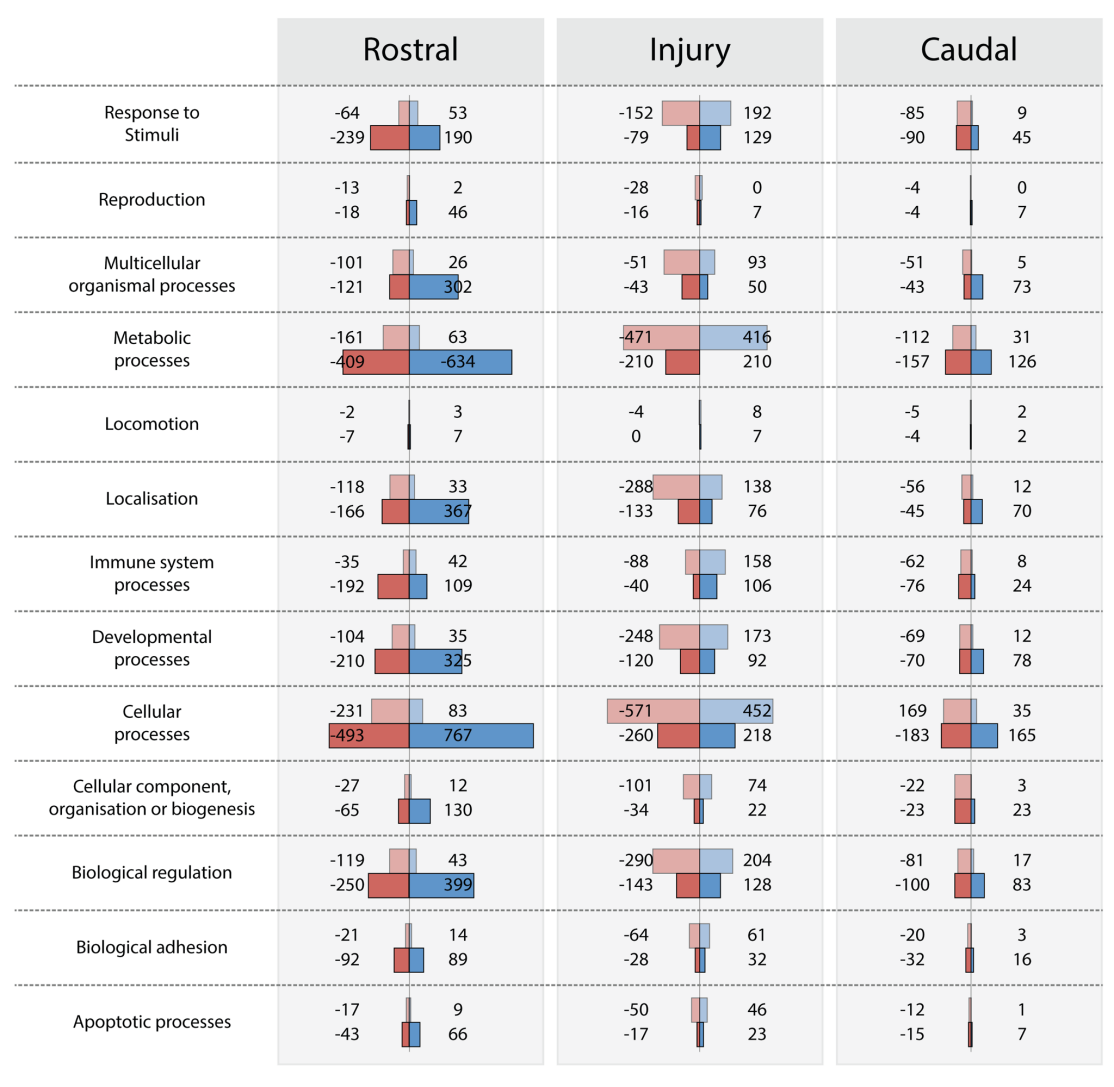
Biological characterization of the significantly (FC>2 or FC<-2) up-regulated (blue; positive) and down-regulated (red; negative) transcripts in response to PcTx1 treatment (‘Panther’ gene classification system, Mi *et al.*, 2013). Separate datasets were obtained from animals with SIS scores of 2.5 (top, lighter bars) and 2.75 (lower, darker bars).

All of the gene transcripts with significant changes (i.e. FC>2 or FC<-2) for the two injury sizes (SIS 2.5 and 2.75) were separated into their biological categories ( Figure 9) using the ‘Panther’ gene classification system ( Mi *et al.*, 2013). Genes classed as cellular (~21%) and metabolic (~18%) processes showed the most changes. Gene changes were also observed for apoptotic (~2%) and immune system (~5%) response – categories of potential importance to tissue preservation following SCI. However, when canonical genes known to be involved in the intrinsic and extrinsic apoptotic pathways ( Elmore, 2007) were analysed individually, no differences between PcTx1-treated and saline-treated rats at any injury severity or in different regions of the cord were detected ( Supplementary file 3). The change in caspase-3 levels barely reached significance (FC 2.2) in the rostral segment of SIS 2.5 PcTx1-treated compared to saline-treated animals. However, as a whole, these results suggest that PcTx1 treatment may not have a major influence on apoptotic pathways at the 24 h post-SCI time point.

Lists of markers for main cellular components of the central nervous tissue were obtained from ( Anderson *et al.*, 2016), see Supplementary file 4. Transcripts for neuronal markers were down-regulated for SIS 2.5 injuries in the rostral segment (rbFOX3 FC -2.8; SYT1 FC -2.3) and up-regulated in the injury segment for SIS 2.75 injuries (rbFOX3 FC 6.6; SYT1 FC 4.5). A similar profile was observed for markers of astrocytes with down-regulation in the rostral segment of SIS 2.5 injured animals (Gfap FC -2.6; Aqp4 FC -3.1; Slc1a2 FC -4.2) and up-regulated in the injury segment of SIS 2.75 injured animals (Gfap FC 2.3; Aqp4 FC 2.5; Slc1a2 FC 3.3). No statistically significant differences were observed for oligodendrocyte markers (Olig1 and Olig2) or for myelin (MAG) at any injury size anywhere along the cord. Similarly, there were no significant differences in microglial markers anywhere along the cord (apart from Trem2 in the rostral segment of SIS 2.5 injured animals, FC 2.2). However, CD68 (a macrophage marker) was significantly up-regulated in rostral regions (FC 6.9 in SIS 2.5 and FC: 2.7 in SIS 2.75), but down-regulated for larger injury sizes in both the injury and caudal segments (SIS 2.75 FC -4.8 at injury centre and FC -2.2 in the caudal segment). CD14 and CCL2 showed similar profiles to CD68. Thus, in contrast to apoptotic pathways, immune responses (either local or systemic) and astrocyte involvement appear to be affected by PcTx1 treatment at 24 h post-injury.

### Temporal pattern of blood-SC barrier (BSCB) disruption

**Figure 10.**
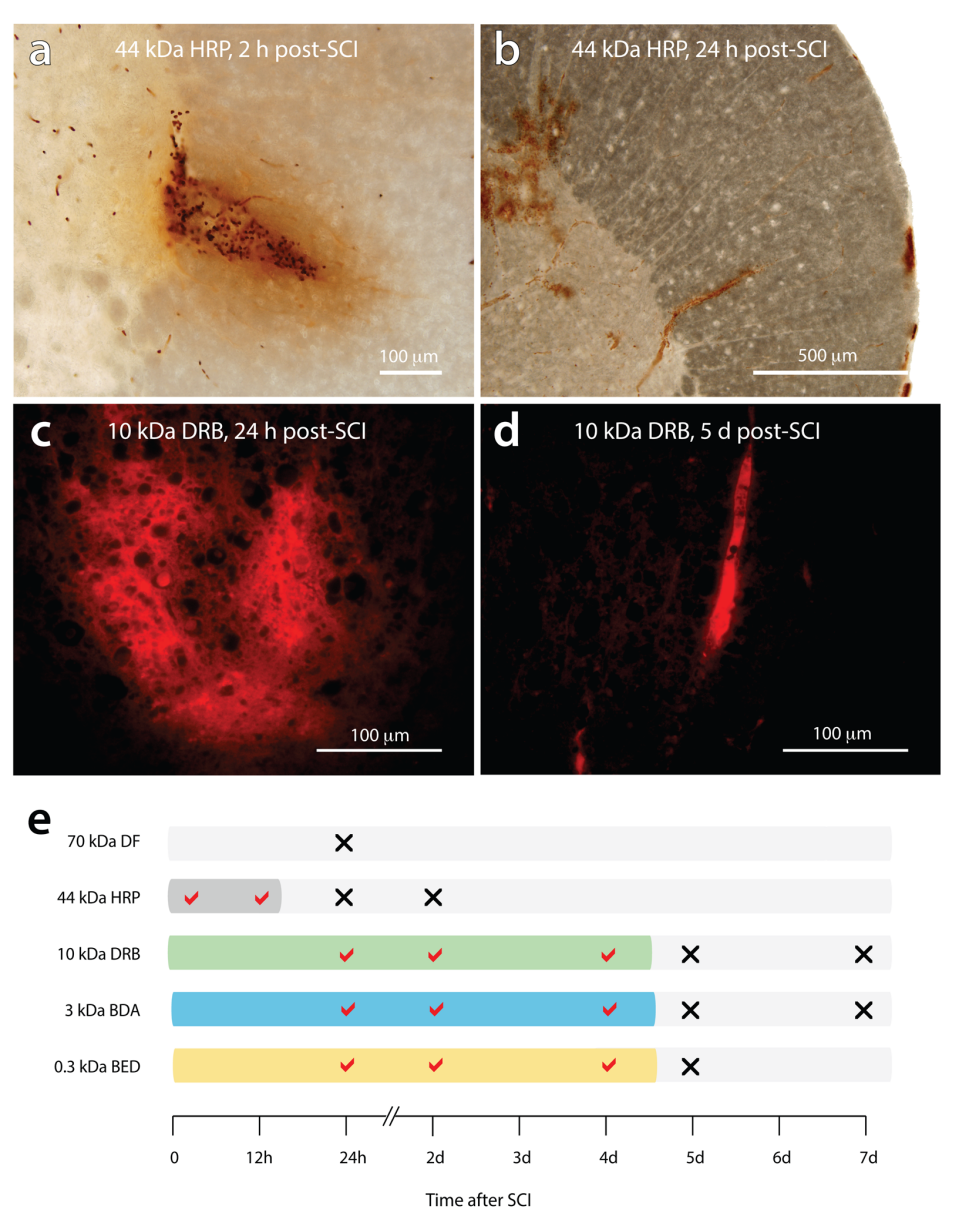
Temporal pattern of blood-spinal cord barrier disruption after SCI. ( **a**) Extravasation of HRP (44 kDa) extending radially out from sites of injury at 2 h post-injury. ( **b**) HRP is confined to the lumen of blood vessels at 24 h post-injury with no visible leakage around sites of injury. ( **c**) Extensive leakage of the 10 kDa dextran rhodamine B is observed around sites of injury at 24 h post-injury. ( **d**) at 5 days post-injury, 10 kDa dextran rhodamine B was always confined to the lumen of blood vessels. ( **e**) diagrammatic summary of the period of blood-spinal cord barrier disruption after SCI vs the molecular size of the permeability tracers. The tracers; dextran fluorescein, 70 kDa DF; horseradish peroxidase, HRP 44 kDa, dextran rhodamine B (DRB 10 kDa), biotin-dextran-amine (BDA 3 kDa), biotin-ethylene-diamine (BED 0.3 kDa) were injected systemically 20 minutes prior to collection of tissue. A tick mark indicates the presence of visible tracer extravasation whereas a cross indicates that the tracers were confined to the lumen of blood vessels.

The different size permeability tracers exhibited different temporal patterns of BSCB dysfunction after SCI ( Figure 10e). At 2 h and 12 h post-SCI, HRP (44 kDa) was observed diffusing radially out from regions of tissue damage into surrounding tissue ( Figure 10a). At 24 h post-SCI, both HRP and the larger dextran fluorescein (70 kDa) were only visible within the lumen of intact blood vessels (see Figure 10b), indicating early restoration of BSCB function for large permeability tracers. All three of the smaller permeability tracers (up to 10 kDa in size) were observed outside blood vessels in and around the injury centre at 24 h, 2 days and 4 days post-SCI ( Figure 10c). By 5 days post-SCI, BSCB function was restored for the smaller tracers with all confined to the lumen of blood vessels, and the lesion core, and no evidence of diffusion out into surrounding tissue ( Figure 10d). 

## Discussion

In this study we investigated whether ASIC1a inhibition improves functional outcomes after traumatic SCI. We also determined the temporal pattern of blood-spinal cord barrier (BSCB) disruption after SCI to define the length of the “ *treatment window*” when there is unrestricted access into the spinal cord. Transcriptomic analysis of spinal cord tissue at the injury centre and in adjacent rostral and caudal regions was performed to better understand the cellular pathways involved in PcTx1-mediated neuroprotection. Taken together, our data indicate that (1) the severity of the initial injuries directly influence long-term functional outcomes and (2) blockade of ASIC1a using a systemically administered peptide inhibitor significantly improves functional outcomes over a range of initial injury severities.

## Blood-spinal cord barrier dysfunction

PcTx1-mediated inhibition of acid-sensing ion channels on spinal neurons and glial cells is crucially dependent on the peptide being able to access spinal cord tissue. Our data show there is a post-trauma “ *treatment window*” for drug delivery that is inversely related to the size of the tracer. For compounds up to 10 kDa in size, the “ *treatment window*” allows 4 days of unrestricted access to spinal cord tissue after SCI. Thus PcTx1 (4.6 kDa) would have been able to access spinal tissue for the entire 48 h treatment period investigated. In this study, dextran rhodamine B (10 kDa) was used as a surrogate tracer for the smaller PcTx1 because it can be directly visualized in fixed tissue sections and avoids the effects that labelling of PcTx1 would have on its physicochemical properties, pharmacological activity, solubility profile and bio-distribution *in vivo*. In addition, our data show that BSCB disruption is highly localised to sites of injury, thus delivering PcTx1 directly to where it is needed ( Figure 10).

### Choice of injury model for assessing neuroprotection

In order to assess efficacy of treatments targeted at reducing the secondary expansion of tissue damage in the central nervous system, it is essential to use experimental models in which secondary tissue loss is a prominent feature. This requirement generally means using small to medium sized lesions as more severe primary lesions that occupy most or all of the cross sectional area of the cord leave little surrounding undamaged tissue into which secondary expansion can occur. For example, the absence of any significant neuroprotective effect in a large animal trial of MgPEG ( Streijger *et al.*, 2016), previously shown to be neuroprotective in rodents ( Kwon *et al.*, 2009), is probably due to the absence of any viable tissue to protect at the injury centre. In the present study, we used controlled impacts to the exposed thoracic spinal cord to produce moderate spinal cord lesions that are initially confined to the central grey matter. Thoracic level injuries enable hind limb function to be used as an assessment of preservation of white matter locomotor tracts. Lower level primary injuries involving lumbar grey matter will cause the loss of lower motorneurons that connect between the spinal cord and individual hind limb muscles. Without lower motorneuron connections, preservation of upper motorneurons in white matter tracts will not be functionally detectable.

### Initial injury severity scoring

To reliably assess the therapeutic effectiveness of treatments to limit secondary tissue loss after SCI, it is essential that comparisons are made between animals that started with similar sized initial injuries, otherwise it cannot be determined if smaller lesions in treated animals at later time points are due to the treatment effect or smaller initial injuries in those animals. Most experimental impactor devices give control over one or more aspects of the impact (force, velocity, depth of tissue penetration and length of compression). It is generally assumed that uniform impacts will produce uniform initial injuries, however this has not been well investigated. The results obtained in this study using highly reproducible contusion impacts to the spinal cord (0.30 m/s ± 0.04 S.D. velocity, 1.44 mm ± 0.01 S.D. penetration, 0.997s ± 0.004 S.D. compression time) showed marked differences in the severity of initial functional deficits between animals (SIS range 1.9–2.75, Figure 2a), scores that reflect marked differences in animals’ abilities to flex hind limb joints ( Table 2). The reason for this variance is likely to be due to inter-animal differences in the number and location of blood vessels that are damaged by the impact, the extent of haemorrhage that occurs and the size of the ensuing region of downstream hypoperfusion. Thus caution needs to be exercised when using aggregate analysis to compare group means between treated and untreated SCI animals. Assessing the severity of functional deficits (or MRI assessments of the extent of tissue damage) soon after injuries are made enables treatment effects to be followed over time within individual animals. The SIS grading scale developed in this study used a small number of simple yes/no objective criteria (does the animal show hind limb weight support, how many hind limb joints can it extend and flex, Table 2), yet yielded very accurate results. Larger initial SIS scores were predictive of greater total amounts of tissue loss ( Figure 5) and poorer residual functional ( Figure 2a) by 6 weeks post-injury. Early assessment of hind limb function has only been employed in a small number of previous SCI studies ( Anderson *et al.*, 2016; Herrmann *et al.*, 2008), but given the variability of lesion sizes observed using highly standardised impacts, we would encourage routine inclusion of initial injury severity measurements in future SCI studies.

### Hind limb functional assessments

Hind limb function is not exclusively driven and modulated by inputs from the brain. Proprioceptive and cutaneous sensory inputs have been shown to be able to drive local motor pattern generators in the spinal cord and generate hind limb stepping locomotion in the absence of any spinal cord connections to or from the brain ( Grillner & Wallen, 1985; Rossignol, 1996). However, these local inputs are greatly reduced, if not absent, when swimming and this provides a useful objective test for the presence of descending supraspinal drive of hind limb locomotor function ( Saunders *et al.*, 1998; Wheaton *et al.*, 2011). All of the SCI animals (PcTx1-treated and saline-treated) were able to swim using alternating kicks of their hind limbs when placed in a tank of water indicating preservation of some supraspinal drive to the lumbar motor centres of the hind limbs. If no functional connections between the brain and lumbar motor centres are present, then differences in hind limb function would not be reflective of preservation of descending white matter tracts.

Most of the locomotor function tests used in this study indicated greater preservation of function at 6 weeks post-injury in the PcTx1-treated animals. The BBB locomotor analysis showed a highly significant improvement in hind limb function of approximately 2 points on the BBB scale in the PcTx1 treated animals ( Figure 2a) that was apparent across the entire range of injury severities investigated. Although that is a modest increase numerically, the effect on locomotor function can be disproportionately large. An increase from 11 to 12 on the BBB scale, for example, corresponds to the difference between the presence and absence of fore limb–hind limb coordination. The PcTx1-treated animals also exhibited fewer foot faults in the horizontal ladder test compared to saline-treated equivalents indicating improved ability to perform coordinated motor tasks following SCI. Two animals with very high SIS scores (one PcTx1-treated and one saline-treated) had many more foot faults than the other rats ( Figure 2b). As an SIS score of 3 indicates complete paralysis (and therefore more extensive initial trauma), it is not surprising that animals with scores close to this would perform poorly on the ladder test.

In contrast to results from the BBB and ladder tests, PcTx1 treatment did not improve motor function on the tapered beam test. A range of factors may have contributed to this. The animals making the most foot faults had a wide range of SIS scores suggesting that walking with legs pressed close together under the body when on the thinnest part of the beam might be difficult for rats of any injury severity. It was also apparent that, after a few trials, many rats learnt that they could walk along the beam using the lower counting ledge without falling and did not even attempt to stay on the narrow beam in subsequent trials. This made it difficult to distinguish between voluntary and involuntary use of the error-counting ledge.

### Tissue preservation

A striking feature of the present study was the complete absence of the central grey matter and large amounts of surrounding white matter at the injury centre ( Figure 4). Immunohistological analysis of serial sections spanning the length of the injured cord segment revealed no significant differences in the number of FOX3 positive neurons between the PcTx1-treated and saline-treated animals at either 24 h or 6 weeks post-injury. In addition, there was no significant change in the number of FOX3 positive neurons between 24 h and 6 weeks which is consistent with our earlier study showing that trauma-induced secondary loss of neurons in the central grey matter is largely complete by 24 h post-injury ( Ek *et al.*, 2010; Ek *et al.*, 2012).

CNPase staining of white matter also showed no significant differences in total white matter area between the PcTx1-treated and saline-treated animals at 6 weeks post-injury despite the significant improvements in locomotor function that were observed in these animals. We investigated further whether the improvements in hind limb function might be due to greater preservation of individual descending white matter tracts that are known to be involved in motor function. A study using injections of biotinylated dextran amine into the motor cortex of mice ( Steward *et al.*, 2004) highlighted three main regions of descending corticospinal motor tracts; at the base of the dorsal column (dcCST), in the dorsolateral white matter on the ventral side of the dorsal horns (dlWM) and in the ventromedial white matter (vmWM) either side of the sulcal fissure. In the present study, the dcCST which normally carries the majority of the descending motor fibres ( Kathe *et al.*, 2014; Steward *et al.*, 2004) was completely absent at 6 weeks post-injury ( Figure 1 & Figure 4) in all of the PcTx1-treated and saline-treated animals. Since all of these animals were able to swim using their hind limbs and recorded high BBB scores this suggests that the dcCST is not essential for driving coordinated hind limb motor function. Detailed analysis of the dlWM region using three separate histological stains (LFB, CNPase and H&E) on adjacent slides from the injury centre showed a greater preservation of white matter in the PcTx1-treated animals compared to saline-treated controls at 6 weeks post-injury ( Figure 5). The total area of preserved dlWM showed an inverse correlation with initial injury severity scores ( Figure 5) and a positive correlation with BBB functional scores ( Figure 6a). 

Preservation of tissue in the vmWM region that normally contains reticulospinal tracts was highly variable and there were no apparent correlations between white matter area in this region and any of the behavioural tests or with the severity of the initial injuries as measured by SIS score.

There was however a noticeable difference in the appearance of the myelin within this region between the treatment groups ( Figure 4). In PcTx1-treated animals, there was an improvement in the quality of the myelin and a decrease in the amount of axotomised axons ( Figure 4). Taken together, these results suggest that it is preservation of axons in the dlWM that is mainly responsible for the improvements in hind limb function in the PcTx1-treated animals.

### Mechanism of neuroprotection

RNAseq differential expression analysis revealed a number of genes whose transcript levels were significantly altered in the PcTx1-treated SCI animals compared to saline-treated SCI animals. Subsequent analysis of transcript variance of the up- and down regulated genes also revealed marked differences in transcript numbers between animals with different injury severities ( Figure 7). Animals with less severe injury severities ( *e.g.* SIS 1.9) exhibited much lower gene transcript numbers for the up-regulated genes and much higher transcript numbers for the down-regulated genes when compared to animals with more severe injuries ( *e.g.* SIS 2.5). This may be due to different magnitudes of the biological responses to injury sizes (such as inflammation levels) that are sensitive to PcTx1. It might also be due to greater vascular disruption in the larger injuries allowing greater extravasation of PcTx1 into the injury site. These pronounced effects of injury severity on gene expression and hind limb functional performance highlight the need to conduct early post-surgery analysis of injury severity in order to compare animals with very similar initial injuries. Aggregating SCI data to conduct parametric statistical analysis using group means is likely to increase the incidence of type 2 statistical errors.

**Figure 11.**
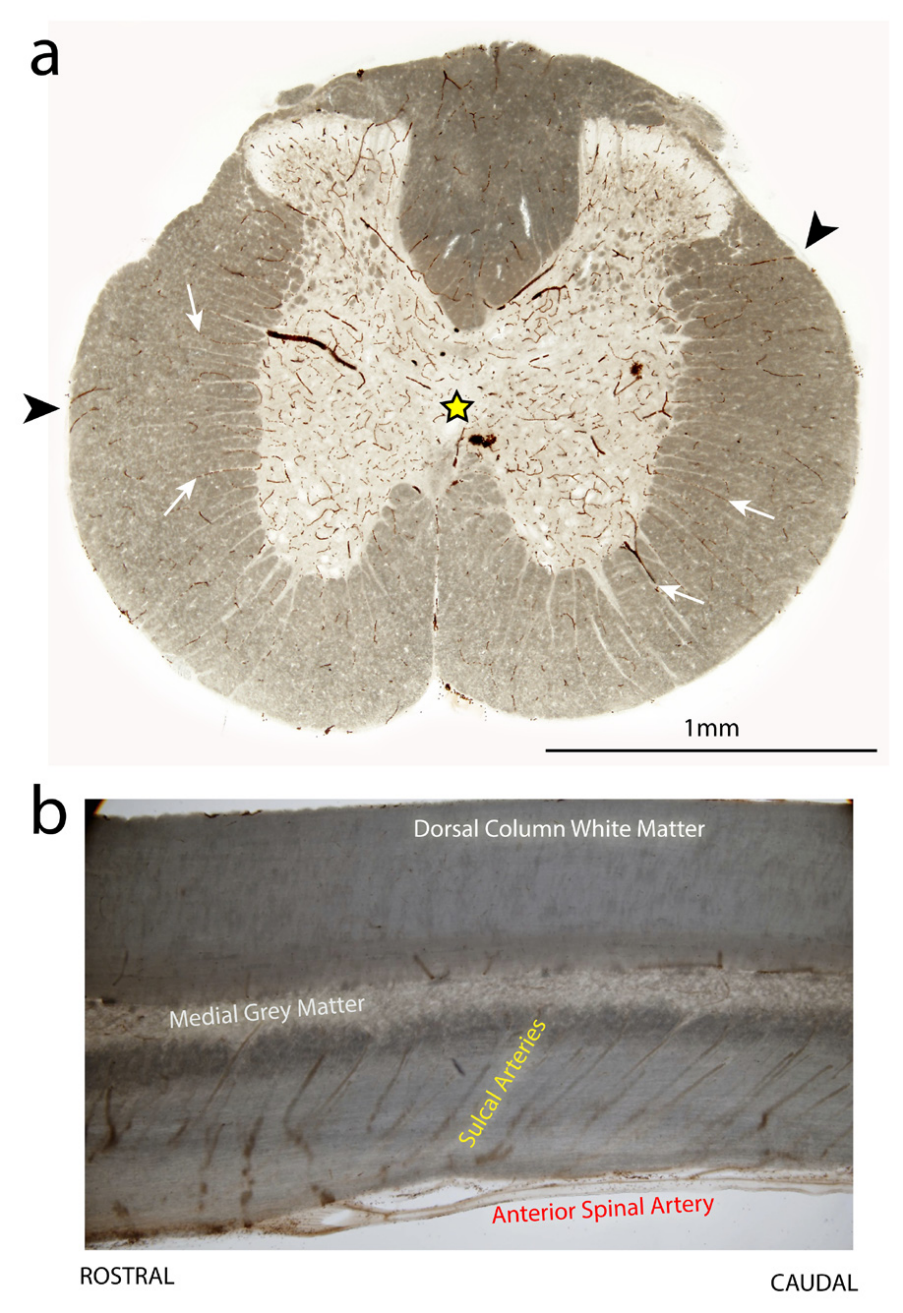
Distribution pattern of blood vessels in the thoracic spinal cord of an uninjured adult rat. Vibratome sections (70 μm thick) from the T10 spinal level and stained with DAB ^+^ kit (DAKO) to highlight blood vessels. ( **a**) is a transverse section. Note the higher blood vessel density in the central grey matter region (yellow star). The deeper layers of the surrounding white matter are supplied by blood vessels originating from the central grey matter (white arrows), whereas the outer layers of white matter are supplied by radial blood vessels penetrating in from the pial surface (arrowheads). ( **b**) is a longitudinal section through the centreline of the cord. Note the rostral to caudal angle of the sulcal arteries which branch off from the anterior spinal artery to supply the central grey matter.

Unique profiles were also observed between the different cord regions (rostral, injury centre and caudal) in terms of the number and complement of genes that changed their expression levels ( Figure 8). This may partly reflect different pathological processes in the different regions, ‘primary’ necrotic damage localized to the injury centre ( Ek *et al.*, 2012) and secondary ischaemic damage away from the injury centre. Another contributing factor could be the predominantly rostral to caudal direction of arterial blood flow at the T10 level of the spinal cord and the rostral to caudal angular orientation of sulcal arteries supplying the central grey matter ( Figure 11b). Thus disruption of grey matter vessels at the injury site might result in less perfusion (and consequent greater hypoxia ischaemia) below the injury site compared to higher rostral segments ( Ek *et al.*, 2010). These results highlight that the pathophysiology of SCI is not homogeneous along the injured cord segment and that neuroprotective treatments may exhibit different efficacies in different areas of the spinal cord and for different injury severities.

PcTx1 treatment did not significantly alter the expression of genes for myelin or oligodendrocytes, which is in contrast to the histological results showing greater preservation of myelin. Similarly, down-regulation of neuronal markers in PcTx1-treated SIS 2.5 animals in the rostral segment and up-regulation in the injury segment in PcTx1-treated SIS 2.75 animals was also at odds with the histological results. The discrepancy between these results may highlight that the main transcriptomic changes occurred at different time-points to the protein changes. It could also mean that individual animals respond to treatment and injury at varying speeds within the first few days.

An interesting observation was an inverse relationship between expression of neuronal and inflammatory markers. In the rostral region of PcTx1-treated SIS 2.5 animals, transcript numbers for neuronal and astrocyte markers were significantly decreased, whilst transcript numbers for the inflammatory markers CD68, CCL2 and CD14 were significantly increased. Conversely, transcript numbers for neuronal and astrocyte markers were significantly increased at the injury centre in PcTx1-treated SIS 2.75 animals, whilst transcript numbers for the three inflammatory markers were significantly decreased. Attenuation of the enriched immune system related pathways (such as CCL2 and CD14) has been proposed as a potential treatment for SCI ( Wen *et al.*, 2016). Our results suggest that PcTx1-mediated effects on the immune response may be an integral component for functional recovery following SCI.

PcTx1 also affected expression of genes encoding for smooth muscle related proteins. This was particularly apparent at injury centre in animals with SIS 2.5 injuries ( Supplementary file 2). Due to the prominent role vascular disruption plays in spinal injury severity, alterations in blood flow might contribute to the effects observed from PcTx1. Future investigation into this topic would be beneficial to the field.

It has been proposed that the neuroprotective effects of ASIC1a inhibitors may be due to prevention of activation of the intrinsic apoptotic pathway ( Friese *et al.*, 2007; Gao *et al.*, 2005; Xiong *et al.*, 2004; Yermolaieva *et al.*, 2004). It has been postulated that acidosis in areas of acute tissue ischaemia opens ASIC1a channels allowing the influx of Na ^+^ ad Ca ^2+^ into neurons and glia. This is thought to cause mitochondrial membrane depolarisation, release of cytochrome c and subsequent activation of intrinsic apoptotic cell death pathways ( Friese *et al.*, 2007; Sherwood *et al.*, 2011; Smaili *et al.*, 2003). A review by Elmore (2007) describes the key proteins involved in the intrinsic, extrinsic and final execution apoptotic pathways. We did not observe down-regulation of any of the key genes in these apoptotic pathways in the PcTx1-treated animals at 24 h post-SCI ( Dataset 1, Table S3). Thus the neuroprotective effects of PcTx1 may not be mediated by a modulation of apoptotic cell death. There remains, however, the possibility that PcTx1 could inhibit apoptotic pathway activation at earlier stages of tissue damage prior to 24 h post-SCI. Previous research in rats has shown increased expression of many inflammatory genes (e.g. IL1β, IL6, MIP2 and MIP1α) in the initial 6–12 h post-SCI before declining to values approaching control levels between 24 h and 48 h ( Carmel *et al.*, 2001). As the transcriptomic analysis in this study was only conducted at a single time point (24 h), there remains the possibility that PcTx1 has even greater effects on earlier phases of the inflammatory response to SCI.

### Concluding remarks

In the spinal cord, “ *tissue is function*” and it is widely accepted that the extent of functional losses after SCI reflects the extent of tissue loss (injury size). It remains to be determined why PcTx1 appeared to primarily preserve white matter (or myelin levels) in the dlWM regions and not in other myelinated tracts within the cord. One possibility is that the model of injury used in this study (central dorsal contusion) disproportionately affects some regions of the cord compared to others. The central grey matter and vmWM regions, for example, lie immediately under the impact site whilst the dlWM is to the side and less directly impacted. Another possibility is that there could be important structural differences between the different areas of the cord. The dlWM may contain a greater proportion of blood vessels originating from the outer pial surface, which would retain greater perfusion, compared to areas with blood vessels originating from the central sulcal (grey matter) supply ( Figure 11a). In order to reveal whether there is evidence of preservation in other areas not visible at the light microscope level, higher resolution analysis of white matter structure at the EM level would be required. For example, greater numbers of myelin sheath wrappings around axons could explain some of the difference in myelin integrity observed between PcTx1-treated and saline-treated animals seen in the vmWM ( Figure 4).

Transcriptomic analysis highlighted several possible mechanisms for the neuroprotective effects of PcTx1 after trauma. Whilst PcTx1 did not appear to affect apoptotic cell death pathways at 24 h post injury, it did alter expression levels of genes involved in inflammatory and immune responses. Further transcriptomic analysis at earlier time points may lead to a better understanding of the mechanisms involved and their timing. In conclusion, this study shows that PcTx1 is effective at preserving white matter tracts involved in hind limb function and thereby improving behavioural outcomes following SCI in the rat.

Raw transcript data for PcTx1- and saline-treated adult rats 24 h after spinal cord injury Raw gene transcript data (Illumina Platform) for PcTx1-treated (n=3) and saline-treated (n=3) adult rats 24 h after mid-thoracic spinal cord injury. The top row shows the injury severity (SIS) score for each animal during the first 24 h post-injury. The second row shows the animal IDs, treatment groups and spinal cord regions. Column a = geneid. Columns b, c, d = rostral segments from saline-treated animals 1, 2, 3. Columns e, f, g = rostral segments from PcTx1-treated animals 4, 5, 6. Columns h, i, j = injury centre segments from saline-treated animals 1, 2, 3. Columns k, l, m = injury centre segments from PcTx1-treated animals 4, 5, 6. Columns n, o, p = caudal segments from saline-treated animals 1, 2, 3. Columns q, r, s = caudal centre segments from PcTx1-treated animals 4, 5, 6. Click here for additional data file. Copyright: © 2016 Koehn LM et al. 2016 Data associated with the article are available under the terms of the Creative Commons Zero "No rights reserved" data waiver (CC0 1.0 Public domain dedication).

## Data availability

The data referenced by this article are under copyright with the following copyright statement: Copyright: © 2016 Koehn LM et al.

Data associated with the article are available under the terms of the Creative Commons Zero "No rights reserved" data waiver (CC0 1.0 Public domain dedication). 

F1000Research: Dataset 1. Raw transcript data for PcTx1- and saline-treated adult rats 24 h after spinal cord injury, 10.5256/f1000research.9094.d128164 ( Koehn *et al.*, 2016).

## Supplementary material

**Supplementary file S1. PcTx1-induced fold changes (FC) in gene expression after SCI.**

Lists of genes with FC >2 or FC <-2 changes in transcript counts at 24 h post-SCI in response to PcTx1 treatment in the rostral (Table S1a), injury centre (Table S1b) and caudal spinal cord segments (Table S1c).

.

**Supplementary file S2. Functional protein association networks for gene changes in PcTx1-treated animals after SCI.**

Functional protein association networks of transcripts that were up-regulated or down-regulated in the injury segment of PcTx1-treated animals compared to saline-treated animals. ( **a**) SIS 2.5 and ( **b**) SIS 2.75 animals. Connecting lines indicate associations between the protein products as determined by the ‘String’ network system ( Szklarczyk *et al.*, 2015). Transcripts were obtained from RNAseq (Illumina).

.

**Supplementary file S3. Effects of PcTx1 on apoptotic pathway expression after SCI.**

Fold changes (FC) of transcript counts for canonical genes in the intrinsic, extrinsic and converging execution apoptotic pathways at 24 h after SCI. Positive values indicate that transcript counts were up-regulated in PcTx1-treated animals compared to saline-treated animals and negative values indicate lower transcript counts for the PcTx1-treated animals. Lists of genes were obtained from Elmore (2007). Transcript counts were obtained from RNAseq (Illumina).

.

**Supplementary file S4. Effects of PcTx1 on expression of cell specific markers after SCI.**

Fold Fold changes (FC) of transcript counts of genes for cellular markers for astrocytes, neurons, oligodendroglia, microglia and endothelial cells at 24 h after SCI. Positive values indicate that transcript counts were up-regulated in PcTx1-treated animals compared to saline-treated animals and negative values indicate lower transcript counts for the PcTx1-treated animals. Lists of genes were obtained from Anderson *et al.* (2016). Transcript counts were obtained from RNAseq (Illumina).
