# Supplementary figures and images for: Selective inhibition of ASIC1a confers functional and morphological neuroprotection following traumatic spinal cord injury
Source: F1000Res. 2016 Dec 7;5:1822. Originally published 2016 Jul 26. [Version 2] doi: 10.12688/f1000research.9094.2 (PMC5200949; doi:10.12688/f1000research.9094.2)

a

SIS 2.5

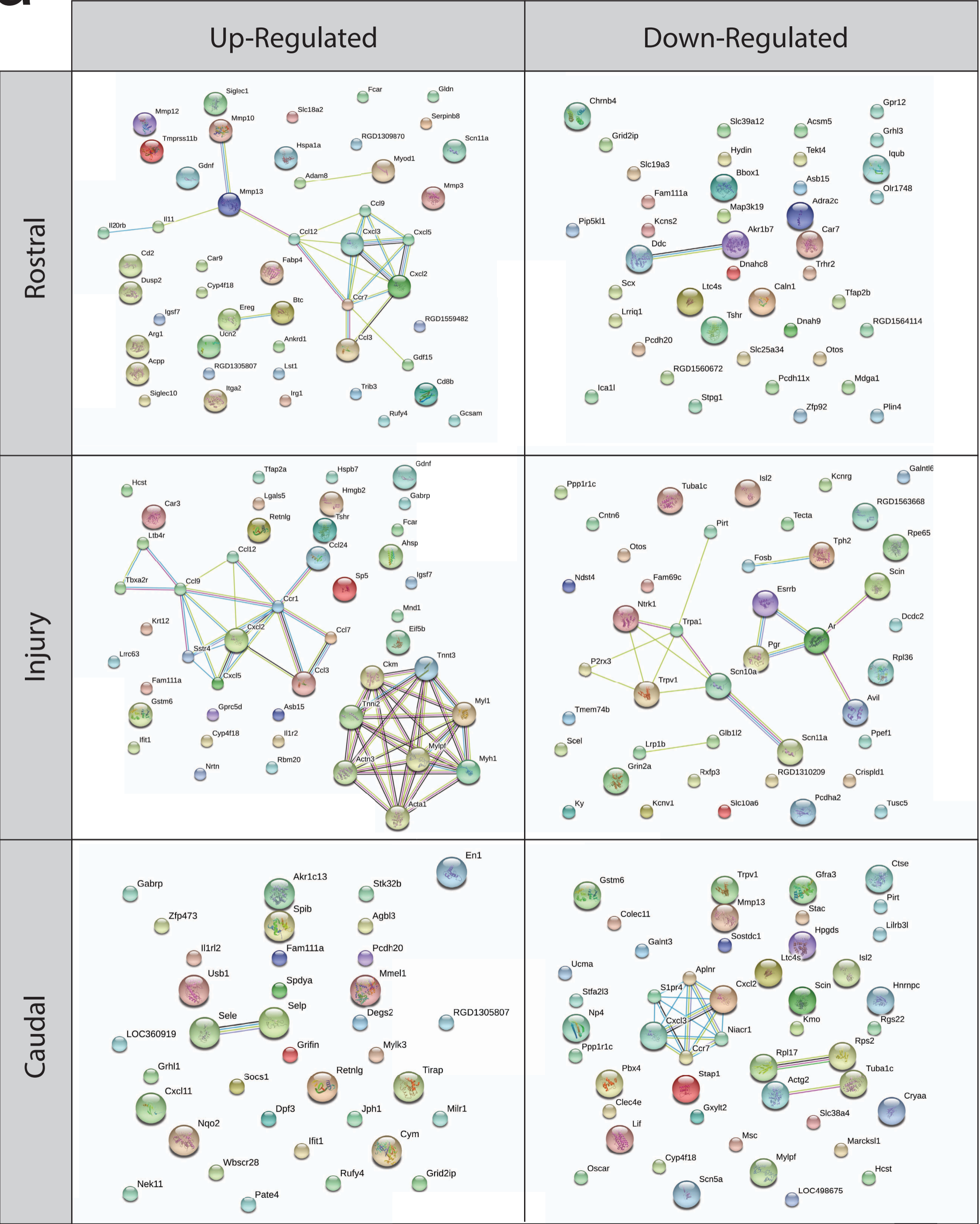

b

SIS 2.75

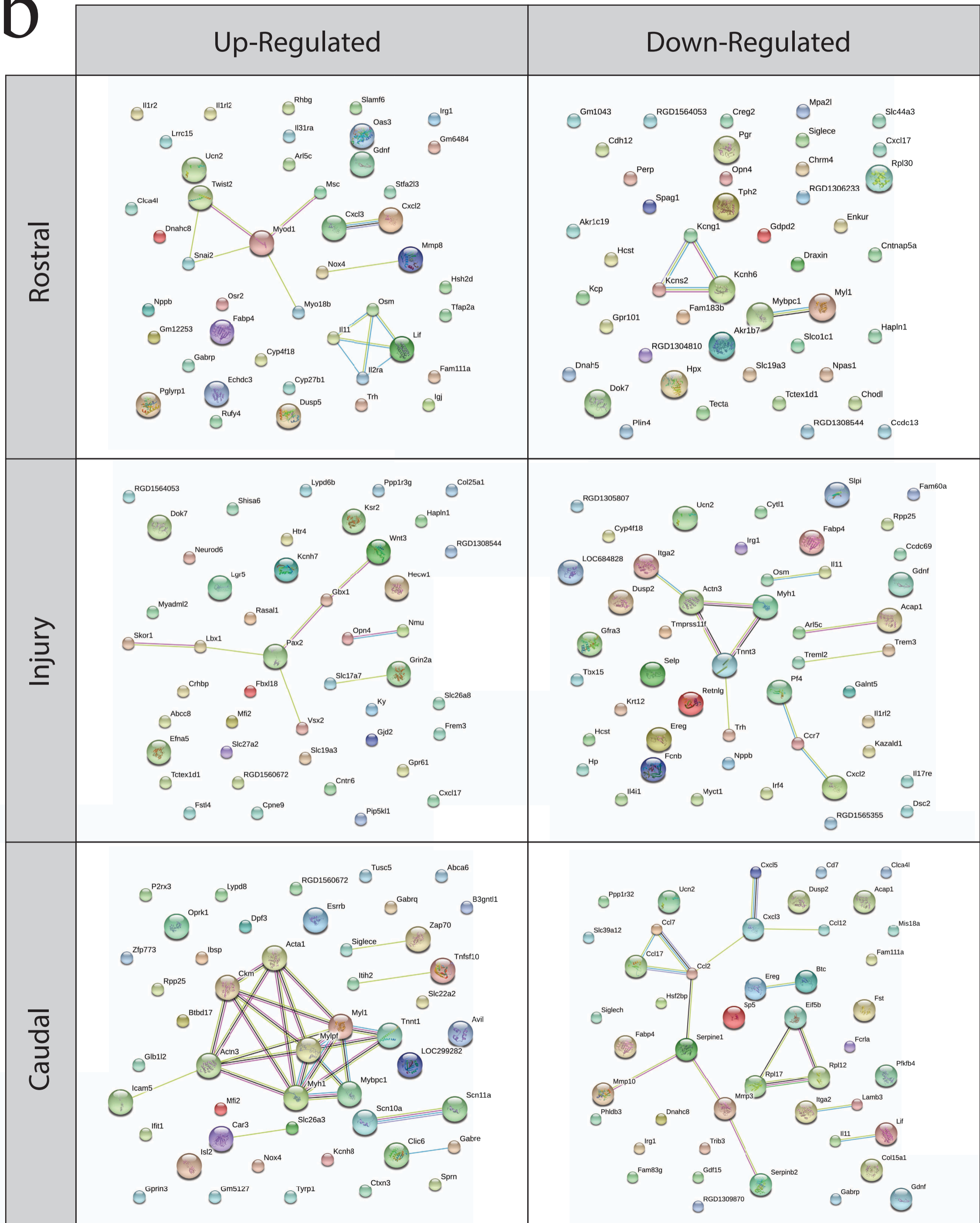

Supplement: Supplementary file 3 [file f1000research-5-11107-s0002.tgz › da8efc53-4197-41ef-aa08-4a78ed42ab27.pdf]
