# Supplementary material for: Selective inhibition of ASIC1a confers functional and morphological neuroprotection following traumatic spinal cord injury
Source: F1000Res. 2016 Dec 7;5:1822. Originally published 2016 Jul 26. [Version 2] doi: 10.12688/f1000research.9094.2 (PMC5200949; doi:10.12688/f1000research.9094.2)
Supplement: Peer review history

# Peer review: Referee response for version 2

**DOI:** 10.5256/f1000research.11107.r18324

**Published:** 2016-12-19

**Authors:** David Magnuson, Darlene Burke

**Affiliations:** 1 Kentucky Spinal Cord Injury Research Center, University of Louisville, Louisville, KY, USA

The authors were responsive to our concerns and either made appropriate changes or provided well reasoned explanations for why they chose not to. The paper is improved and in particular the description of the statistical analysis and use of the F statistics are now much easier to follow.

We have read this submission. We believe that we have an appropriate level of expertise to confirm that it is of an acceptable scientific standard.

# Peer review: Referee response for version 1

**DOI:** 10.5256/f1000research.9788.r15249

**Published:** 2016-09-30

**Authors:** Helen Marie Bramlett

**Affiliations:** 1 Department of Neurological Surgery, University of Miami, Miami, FL, USA

This manuscript reports on the use of a novel injury severity score for spinal cord injury to determine treatment efficacy on histological, functional and transcriptomic outcome measures. The treatment chosen was a selective inhibitor of ASIC1a, PcTx-1. The authors compared extensively through correlation how PcTx-1 improves function as well as white matter preservation based on the individual animal’s severity score.  Overall, this treatment appears to improve several outcome measures but did not significantly impact the transcriptome analysis targeting the apoptosis pathway. However, the use of the injury severity score has merit when determining drug efficacy because individual animals are analyzed based on this score. When using this type of analysis, the heterogeneity of SCI can be taken into consideration.

**Specific Comments:**

1. Overall, this is a well-written manuscript. However, several minor points are at issue.
2. The authors state that the use of the osmotic pump for PcTx1 release resulted in stable plasma concentrations of the drug over a 48 hr period, how was this determined? Were levels taken or was this an assumption?
3. In using the beam test, animals are usually pretrained on this task but there is no mention of this in the methods section.

I have read this submission. I believe that I have an appropriate level of expertise to confirm that it is of an acceptable scientific standard.

# Response

**Published:** 2016-11-22

**Authors:** Mark Habgood

**Affiliations:** University of Melbourne, Australia

*"The authors state that the use of the osmotic pump for PcTx1 release resulted in stable plasma concentrations of the drug over a 48 hr period, how was this determined? Were levels taken or was this an assumption?"*

 Yes this was an assumption.

 The role of the pump was to slowly and steadily release PcTx1 (1.08 μg/h) subcutaneously to compensate for estimated renal and tissue losses over a 48 h period.

 PcTx1 is a cysteine knot peptide which makes it very stable and resistant to plasma peptidases with a half-life >12 hours (unpublished). Thus, *in vivo*, the main plasma losses are due to renal excretion

*"Pre-training"*

 The animals were pre-trained on the beam apparatus prior to commencement of the surgical and treatment protocols. On the day of functional testing, the animals were allowed 3 practice runs prior to recording. This information has been added to the Methods.

# Peer review: Referee response for version 1

**DOI:** 10.5256/f1000research.9788.r15759

**Published:** 2016-08-31

**Authors:** David Magnuson, Darlene Burke

**Affiliations:** 1 Kentucky Spinal Cord Injury Research Center, University of Louisville, Louisville, KY, USA

This is a very well-written paper that clearly describes the fairly complex experimental design and rationale and nicely presents the primary data. The main question addressed is if immediate systemic application of recombinant PcTx1, a spider venom toxin, can reduce tissue damage and improve function in male SD rats given mild contusive spinal cord injuries. All of the primary functional, histological and transcriptomic data were collected and analyzed in a blinded fashion. However, the statistical analysis appears to be unusual for this kind of study and the overall outcome is rather unclear.

 The investigators chose to use a novel SIS (simplified injury severity) score at the time of injury and again at 24 hours post-injury.  The SIS is based on a rather coarse and certainly non-linear 6-point scale (0-3 in 0.5 unit increments). Nonetheless, the 24h SIS score plays a very important role in the paper. Functional assays at 24h, whether they be visual scales like the BBB or video based kinematics, are poor predictors of terminal hindlimb function when moderate to severe injuries are used because most of the animals will exhibit flaccid paralysis at that early time point. In this paper they show that the SIS at 24 hours is predictive of terminal hindlimb function (BBB scores) following the mild injuries employed, even when only part of the scales range is used (1.5 – 2.75).

 The other functional assessments included the BBB Open Field Locomotor Score (without subscore), horizontal ladder and tapered beam tasks and swimming, the last three utilizing video to assist with counting footfalls/slips and to assess hindlimb alternation. Interestingly, the investigators found significant functional differences only using ANCOVA analysis of BBB scores at 6 weeks vs initial injury severity score (SIS). No differences were seen in ladder, tapered beam or swimming, although the pattern of data points is somewhat suggestive that PcTx1 treatment reduced errors on the horizontal ladder.

 This paper also presents a substantial amount of data on gene expression at 24h post-injury using the Illumina platform and marker extravasation at various time points. These data provide a lot of food for thought but unfortunately do not really allow any strong conclusions to be drawn except that the blood spinal cord barrier remains leaky to small markers for up to 4 days after a mild contusive spinal cord injury thus providing a window of pharmacological opportunity wherein small molecules, like PcTx1, may enter the spinal cord from the bloodstream to confer protection.

 Major Concerns:

1. The primary finding of the paper, that PcTx1 improves locomotor function of adult SD rats after thoracic SCI is only partly supported by the data. Traditionally, the BBB and other functional (and hopefully objective) assessments would be done weekly and changes over time would be observed. Group differences in BBB or BBB subscore along with objective kinematic or gait analysis would be used to conclude that a tissue-sparing strategy resulted in functional improvements or it did not. In this case the authors chose to assess the locomotor function at 6 weeks only and to reduce the effect of inter-animal variability using the 24h SIS scores and ANCOVA analysis.
2. Using ANCOVA is unusual in this kind of experiment, but can be done only if the correlation coefficients of the two groups are equal, and this information has not been provided. For each of the regression lines a 95% confidence interval should be shown. For the BBB vs SIS data, it should also be explained what test was done to determine that the PcTx1-treated group had a “significantly higher elevation compared to the saline-treated group”. It is assumed that this is part of the ANCOVA analysis, but more details should be given. Similarly, for the dlWB vs SIS data, it should be explained what test was done to determine significance and 95% confidence intervals should be shown. The outcomes of these tests should also be provided for the other data sets shown in Figures 2 and 6, even if there are no significant differences.

Minor Concerns:

1. In the subsection “Functional Testing” the authors suggest that hindlimb alternation during swimming is indicative of supra-spinal connections without giving a reference. Subsequently, in the results section they indicate that there were no differences in swimming between the groups. This is meaningful only if the analysis was extended beyond the presence or absence of alternation and I believe more information is necessary or perhaps the reference to swimming should be removed as it adds nothing to the paper. We have used swimming extensively in my laboratory and have seen animals with a wide range of spared white matter accomplish hindlimb alternation.
2. If notes were taken during the BBB scoring it should be possible to determine the BBB subscores that have proven to be very helpful in other studies using mild thoracic injuries.
3. In the section “Blood-spinal cord barrier integrity” the authors reference using an overdose of inhaled anesthetic. It is recommended in the AVMA Guidelines on Euthanasia that a secondary method of ensuring death is employed and this should be described here.
4. In this same section it is obvious that an additional cohort (or cohorts) of animals were employed to develop the time course/size of tracer relationship and these animals should be included at the beginning of the methods section to indicate the real total of animals used in the studies.
5. For histological analysis the investigators chose to assess dorsolateral funiculus, ventral white matter and corticospinal tracts using a myelin stain. While this is acceptable, the rationale for these choices includes “white matter tracts involved in hind limb motor function”. While this statement is true, it is not complete. There are many examples in the literature demonstrating that the ventrolateral and lateral white matter are at the top of the hierarchy for hindlimb function. Thus, including the lateral and ventrolateral white matter in the analysis would be ideal, but at the very least the statement should be amended to indicate that the areas chosen are among those involved in hindlimb function after SCI. With this caveat, the investigators have done an excellent job of analyzing spared white matter and pointing out the poor appearance of ventral tracts.
6. Comments in the discussion point to the non-linearity of the BBB scale and suggest that a mean difference of 1 or 2 points may be small (on the scale) but functionally significant. This may be true for certain ranges of the BBB (8-10 or 11-13), but is arguable not true for the range used here (16-18). A more sensitive gait assessment where order and timing of paw placement would have been helpful in this situation.

Figures:

1. The images in Figure 1 have quite different backgrounds which may add unwarranted contrast to the injured image (right). This should be corrected.
2. The indicator of significant differences in Figures 2 and 5 should be made more obvious. Increasing the size and blackness of the font and line/arrows should suffice.
3. The size of the font used in the legends is inconsistent and in some cases is too small (Figure 6).
4. The image in Figure 11b is too small and the text is way too small. 

We have read this submission. We believe that we have an appropriate level of expertise to confirm that it is of an acceptable scientific standard, however we have significant reservations, as outlined above.

# Response

**Published:** 2016-11-22

**Authors:** Mark Habgood

**Affiliations:** University of Melbourne, Australia

*The investigators chose to use a novel SIS (simplified injury severity) score* ***:***

 The injury severity scale was used primarily to provide an index of the degree of initial dysfunction resulting from the contusion injury. In many experimental studies it is generally assumed that uniform mechanical impacts applied to the same spinal cord locations will produce very similar injuries with similar degrees of dysfunction. However, we have found that this is not the case and that the degree of initial dysfunction is much more variable and appears to depend on the extent and location of the initial damage and vascular disruption/haemorrhage .

 The scale was intentionally developed to use hind limb motion features that are readily apparent to an observer and do not require more subjective interpretation (e.g. can the animal weight support it’s hindquarters or not). The scale is undoubtedly non-linear, a feature in common with most locomotor functional scales used in the field (e.g. BBB scale), but it is proportional. Less severe injuries give lower values than more severe injuries.

 The scale does play an important role in the paper and this is a major point that we are trying to emphasise. The inherent variability of the initial injuries needs to be taken into account individually when assessing improvements in functional performance. 

 "Functional assays at 24h, whether they be visual scales like the BBB or video based kinematics, are poor predictors of terminal hindlimb function when moderate to severe injuries are used because most of the animals will exhibit flaccid paralysis at that early time point."

 Temporary flaccid paralysis is much less common after light to moderate injuries where less tissue damage is involved. In our study, none of the animals exhibited flaccid paralysis, all had some residual ability to move some or all of their joints. The scores assessed during the first 24 hours post-injury were found to correlate with long term functional outcomes.

 Less severe injuries were intentionally used in this study because the focus was treatment-induced reductions of secondary tissue damage expansion using hind limb function as one assay of effectiveness. With thoracic spinal cord injuries, it is the white matter tracts that are primarily involved in supraspinal control of hind limb function. With more severe injuries, the primary damage zone often includes large areas of the white matter tracts which leaves little room for radial secondary expansion. In this study, the initial injuries (confirmed by histology at 15 minutes post-SCI) were confined to the central grey matter with little involvement of surrounding white matter tracts (Ek *et al.,* 2010).

**Major Concerns:**

**1.**

 It is well known in the field that recovery of function after spinal contusion injury gradually improves over the course of several weeks until it reaches a plateau. We chose 6 weeks post-injury as being a time point well after the plateau in functional recovery. Our objective was to determine what was the ultimate level of functional recovery that could be achieved following 48 hours treatment commencing immediately after injury (i.e. were their long term functional improvements). The rate of functional recovery was not an objective of the study.

 We agree that kinematic analysis could provide valuable detailed analysis, however this is often not available in many parts of the world. Therefore we wanted to demonstrate that even using simple and easily available techniques that targeting ASIC1a demonstrate significant differences providing the experimental design takes into account the variance of initial injuries.

Use of first 24 hours SIS scores.

 In this study we have shown that almost identical mechanical contusion impacts (see Results for impact velocity, tissue penetration and compression time) result in a variable range of initial dysfunction and thus the assumption that all animals are starting from the same functional level is not correct. Furthermore, we have also shown that the long-term functional outcome of individual animals correlates linearly with the degree of initial dysfunction (i.e. within each group, the differences in initial injuries are contributing to the observed differences in ultimate functional outcomes).  

 Our opinion is that ignoring the presence of a known source of variance in order to use parametric testing will increase the likelihood of making a Type 2 statistical error (i.e. by assuming that the covariate has no influence on the measurements being made).

**2.**

 Tests for equal slope (correlation coefficients) are part of the ANCOVA.

 The ANCOVA software (PRISM 6.0, Graphpad) uses the general linear model approach which fits linear regression lines to the datasets and then tests whether the slopes (correlation coefficients) are similar. If the slopes are confirmed to be the same, differences in the intercepts (elevations) can then be tested. If the slopes are not the same, differences in the intercepts cannot be calculated.

 As part of the statistical analysis, the two data groups being compared (i.e. control and PcTx1-treated) were analysed to determine if they were best described by (a) a single data group (i.e. there were no differences in slopes or intercepts), (b) two separate groups with different slopes or (c) two separate groups with the same slope, but different intercepts. In the text we have used the phrase “the data were best described by two separate lines with the same slope” to indicate that the slopes (correlation coefficients) were found to be the same. We have also added additional statistical information to the figure legends where appropriate (F statistic with degrees of freedom).

 The suggestion of adding 95% confidence intervals is excellent and these have been added to figures where appropriate. 

*“It is assumed the test to determine significantly higher elevations is part of the ANCOVA.”*

 Yes, this is a fundamental part of the ANCOVA. The slopes (correlations) are tested first and if confirmed to be the same, differences in the intercepts (elevations) are then tested. The mathematics involved in the software is an F test. We have revised the figure legends to include the pooled slopes where significant differences are shown, and the F statistics for the comparisons. We have also added the name of the software package used for the ANCOVA (PRISM 6, Graphpad) and further explanation of the ANCOVA method to the Methods section.

 Figure 6a shows raw data for BBB scores vs dorso-lateral white matter area. The data for the PcTx1-treated animals fall on the same general correlation as for the untreated animals, albeit the treated group had larger areas of preserved tissue compared to the untreated group. Initial analysis revealed the data were best described as a single dataset with the same slope and intercept. The figure emphasises the point that BBB performance appears to relate primarily to the amount of preserved white matter in both groups.

 Similarly, Figure 6b shows raw data for horizontal ladder foot faults vs dorso-lateral white matter area. This figure emphasises that number of foot faults made on the horizontal ladder depends on the amount of preserved white matter. **

**Minor Concerns:**

**1.**

 We apologise for omitting the references to the swimming test, these have now been added to the Methods section (Saunders *et al.,* 1998; Smith *et al.,* 2006; Magnuson *et al.,* 2009).     

 The swimming test was included primarily to determine whether there was evidence of voluntary use of the hind limbs (when the limbs are not weight supporting) that would indicate some preservation of supra-spinal control. We have previously shown that opossums with complete spinal cord transections (no remaining supraspinal connections) do not move their hind limbs when swimming, but they do use them when climbing out of the tank and walking over a hard surface (Wheaton *et al.,* 2011).

 All animals in this study used their hind limbs in a brisk alternating pattern similar to uninjured controls.

 BBB subscores were not analysed.

**2.**

 The secondary method approved by our Animal Ethics and Welfare Committee was immediate opening of the chest cavity and transcardial perfuse fixation with paraformaldehyde. This information has been added to the Histological analysis section. 

**3.**

 The 38 animals that were used for the tracer experiments have been added to the Methods section.

**4.**

 Amended as suggested.

**5.** 

 We accept this recommendation which as an additional “less-subjective” assessment could be helpful to confirm treatment-induced improvements in BBB function.

**Figures**

 Suggested changes made.
